# Prevalence and impact of molecular variation in the three-prime repair exonuclease 1 TREX1 and its implications for oncology

**DOI:** 10.1186/s40246-025-00785-y

**Published:** 2025-06-28

**Authors:** Marwa Shekfeh, Mariam M. Konaté, Julia Krushkal

**Affiliations:** Biometric Research Program, Division of Cancer Treatment and Diagnosis, National Cancer Institute, NIH, 9609 Medical Center Dr., Rockville, MD 20850 USA

**Keywords:** TREX1, Sequence variant, Copy number alteration, Innate immunity, Cancer, Autoimmune disease

## Abstract

**Background:**

The three-prime repair exonuclease 1, TREX1, degrades cytosolic DNA to prevent aberrant immune activation. Its inactivation results in DNA accumulation in the cytosol and induction of the cGAS-STING DNA sensing pathway, interferon signaling, and inflammation. Germline pathogenic *TREX1* mutations are known to lead to hereditary autoimmune and autoinflammatory disorders, whereas the consequences of *TREX1* mutations in cancer remain poorly understood.

**Results:**

To assess the importance of human TREX1 amino acid variants, we analyzed protein sequences of the functional TREX1b isoform from 168 mammalian species and integrated available data on TREX1 sequence and copy number alterations in hereditary autoimmune and autoinflammatory disorders, cancer, and in human populations. While the entire TREX1b protein was conserved in placental mammals, egg-laying mammals and marsupials had their own unique C-terminal regions, with each predicted to contain a transmembrane domain. We modeled human TREX1 variants occurring in autoimmune disease and cancer samples at 12 protein positions to evaluate their predicted impact on protein stability and function.

**Conclusions:**

Our findings provide novel insight into the role of TREX1 molecular variation in cancer, where genetic or epigenetic loss of TREX1 activity may improve susceptibility to treatment. However, *TREX1* gene deletion in tumors was associated with unfavorable patient outcomes, most likely due its frequent co-occurrence with the loss of the entire 3p chromosomal arm, which contains known cancer-related genes.

**Supplementary Information:**

The online version contains supplementary material available at 10.1186/s40246-025-00785-y.

## Background

The three-prime repair exonuclease 1, TREX1 (DNase III) is an important regulator of innate immune response in the cell [1–6]. It is a member of the DEDD (DnaQ-like, or DEDDh) family, also known as RNAse T superfamily [7–12]. TREX1 is a 3ʹ-5ʹ repair exonuclease, which degrades single-stranded (ssDNA) and double-stranded DNA (dsDNA) in the cytosol (Fig. S1) [7–12] that may be generated, e.g., by viral infections, endogenous retrotransposon activation, chromosomal damage, loss of cell cycle checkpoint, formation and rupture of micronuclei, or mitochondrial damage [4, 13–16]. TREX1 is a homodimer protein, which, by degrading cytosolic DNA, suppresses the activation of the cGAS-STING innate immune pathway and prevents immune activation mediated by interferon signaling [2, 3, 8, 16, 17]. Human TREX1 is also an exoribonuclease which degrades ssRNA and DNA/RNA hybrids [9, 18]. Through its degradation of nucleic acids in the cytosol, TREX1 serves as an important factor in the control of endogenous retrotransposons, including long interspersed repeats (LINE-1, or L1), endogenous retroviruses, and short interspersed repeats (SINE) [1, 19–21]. TREX1 also facilitates the clearance of micronuclei [21]. In response to DNA damage, TREX1 is upregulated and is translocated to the nucleus, where it interacts with PARP1 (poly(ADP-ribose) polymerase-1); in addition, TREX1 also interacts with and is co-translocated jointly with the SET complex [13–15, 22–24]. Some studies suggested that via generation of ssDNA on chromatin bridges, TREX1 resolves chromatin bridges which may arise during telomere crisis [25, 26].

TREX1 has three protein isoforms, which differ from one another by the presence or absence of additional N-terminal fragments. Isoform b (TREX1b), a 314 aa long polypeptide with molecular weight of ~ 33 kDa encoded by a single exon, has both the highest expression in human cells and the highest activity in DNA and RNA degradation [18, 24, 27]. The shortest isoform c (TREX1c; 304 aa) has a similar DNase activity to TREX1b in vitro, however its RNase activity is reduced [18]. The longest TREX1 protein isoform a (TREX1a; 369 aa), is expressed in microglia of healthy individuals and patients with retinal vasculopathy with cerebral leukodystrophy (RCVL); however, understanding of the functional role of this isoform remains limited [28]. The N-terminus of human TREX1 has 3ʹ-5ʹ- exonuclease activity, whereas its C-terminus contains the transmembrane domain (TMD), which is involved in the tethering to the endoplasmic reticulum (ER), thereby playing a critical role in TREX1 localization to the micronuclei and TREX1-mediated degradation of DNA from the ruptured micronuclei [2, 7, 16, 29–31]. The C-terminus also interacts with the oligosaccharyltransferase (OST) complex on the ER and participates in OST regulation [32, 33].

The human *TREX1* gene is located at 3p21.31 [24, 34]. Its absence, silencing*,* or mutational inactivation lead to accumulation of cDNA in the cytosol, upregulation of the cGAS-STING-mediated interferon response, systemic inflammation, impaired G1/S transition, constitutive activation of ATM-mediated DNA damage checkpoint, and resistance to apopototic cell death [2, 3, 7, 8, 35, 36]. Human germline pathogenic *TREX1* mutations have been associated with a number of hereditary autoimmune and autoinflammatory disorders, including RCVL, Aicardi–Goutières syndrome (AGS), systemic lupus erythematosus (SLE), familial chilblain lupus (FCL), and cerebral autosomal dominant arteriopathy with subcortical infarcts and leukoencephalopathy (CADASIL) [3, 27, 36, 37]. Germline defects or copy number loss of *TREX1* have also been reported in patients with neurodevelopmental disorders other than AGS. They include a pathogenic *TREX1* single nucleotide variant (SNV) in a patient with intellectual disability [38] and a 3.1-Mb microdeletion of the 3p21.31 region including *TREX1* in a patient with cortical blindness, cleft lip, central nervous system abnormalities, and developmental delay [39], although causality of such variants has not been established.

Earlier studies examined phylogenetic relationships and domain conservation of TREX1 and other DEDD exonucleases [7, 11, 18, 21, 40]. In addition to *TREX1*, the human genome also contains its paralog *TREX2*. Its protein has 44% sequence identity with human TREX1, lacks the 68 aa C-terminal extension of TREX1 and does not have exonuclease activity [6, 8, 41]. TREX1 orthologs and paralogs exist in many diverse taxa and include, e.g., bacterial RNAse T [9] and viral DEDD exonucleases, which were acquired by *Nidovirales* viruses including coronaviruses [11]. Although *TREX1* was initially considered to be unique to mammals [42], its orthologs were identified in amphibians and reptiles [21].

While the effects of germline inactivating mutations on TREX1 protein structure, function, and autoimmune disease have been extensively documented [7, 9, 27, 36], understanding of the effects of TREX1 variants in cancer is only now emerging. Through its degradation of cytoplasmic DNA, TREX1 was recently reported to play a protumorigenic role by inhibiting cGAS-STING mediated interferon signaling and enabling chromosomally unstable tumors to escape from immune surveillance [29, 43–46]. Additionally, multiple studies demonstrated associations of reduced mRNA and protein expression and increased DNA methylation of *TREX1* with improved response to anticancer treatment [13, 15, 47–52]. TREX1 is upregulated after treatment of malignant cells with DNA damaging agents, radiation therapy, or UV light exposure, and it attenuates tumor response to chemotherapy and radiotherapy by modulating the activation of immune signaling pathways, which are induced by cytosolic DNA fragments generated by treatment-induced DNA damage [13, 15, 47–50]. TREX1 protein expression is increased in multiple tumors, and higher levels of its expression were associated with worse prognosis in patients with wild type p53, as p53 promotes TREX1 protein degradation [53].

Even though an increasing number of studies have highlighted the importance of TREX1 as a novel target for cancer treatment, understanding of consequences of somatic or germline *TREX1* variants remains limited. The loss or inactivation of TREX1 in cancer cells restricts tumor growth and potentiates checkpoint inhibition of the adaptive immune response [29, 43–45]. The broader 3p21 chromosomal region containing *TREX1* is frequently deleted in cancer, however, most attention has been drawn to the nearby 3p21.3 and 3p21.1 regions, which contain important tumor suppressor genes [54–57]. Despite the importance of *TREX1* in the control of cytoplasmic nucleic acids and interferon-mediated immune activation, little is currently known about the prevalence of its variants in malignant cells and their potential antitumor effects. To better understand the impact of TREX1 variation, there is a strong need for a systemic catalogue of all germline and somatic variants that occur in tumor cells, and for their comparison to the variants in healthy individuals and in patients with autoimmune disease.

Recent genome sequencing projects provided unprecedented amounts of data in phylogenetically distinct placental mammals and within more closely related non-human primates [58–60]. Such technological advances provide opportunities to investigate TREX1 conservation among a large number of mammalian species and to use sequence conservation to fine map functionally important protein domains and individual residues. Additional curated information on germline and somatic human *TREX1* variation in population-based samples, disease specimens, and human cell lines and organoids is available from public resources. They include ClinVar [61], LOVD (Leiden Open Variation Database) [62], and Decipher (DatabasE of genomiC varIation and Phenotype in Humans using Ensembl Resources) [63], which predominantly contain information on germline *TREX1* variants in autoimmune disease patients, COSMIC (the Catalogue of Somatic Mutations in Cancer) [64] and TCGA (The Cancer Genome Atlas Program) [65, 66] with data on *TREX1* variants in cancer cells, and gnomAD (the Genome Aggregation Database) [67] with information on population-based *TREX1* variants. In this report, we describe our integration of their data with analysis of TREX1 protein evolution, conservation of TREX1 variants in a wide range of mammalian species, and modeling of TREX1 protein variants to evaluate their predicted impact on protein stability and function. We compared the impact of TREX1 variants predicted by our modeling to available predictions from two popular protein structure resources, proteome-wide AlphaMissense [68] and cancer-centered COSMIC-3D [69]. We provide an integrated catalog of germline and somatic TREX1 DNA and protein variants and discuss their potential impact on human disease.

## Methods

**Fig. 1:**
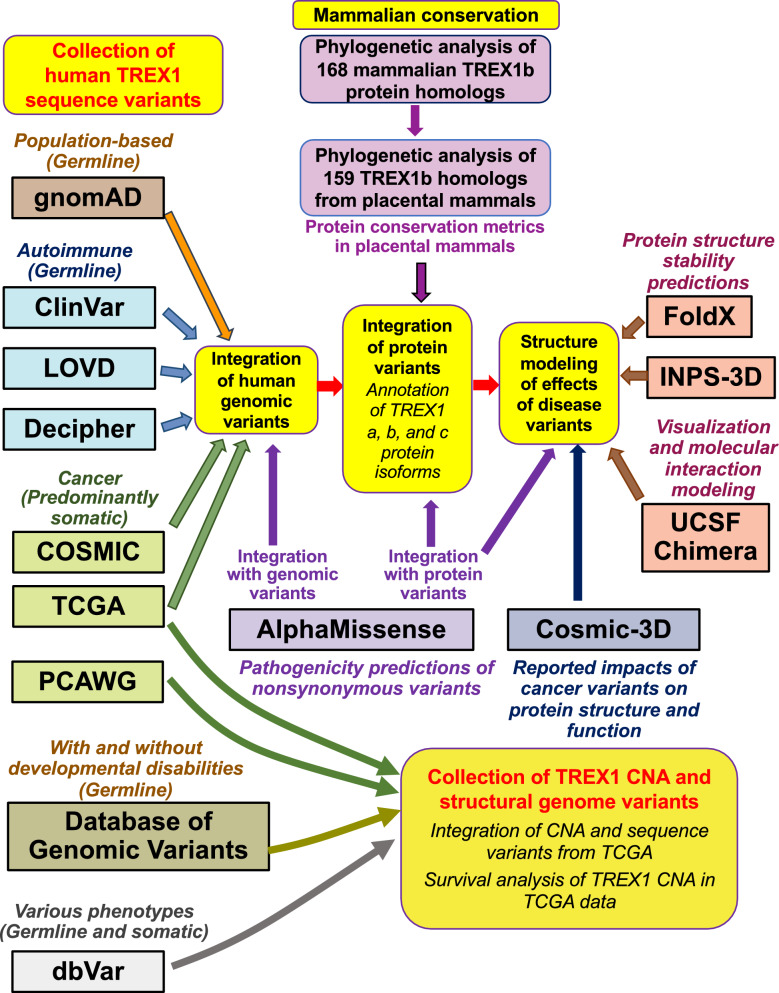
A workflow representing collection, integration, and analysis of TREX1 data. Detailed information about individual steps is provided in the Methods. CNA, copy number alterations; COSMIC, the Catalogue of Somatic Mutations in Cancer; dbVar, database of human genomic Structural Variation; Decipher, DatabasE of genomiC varIation and Phenotype in Humans using Ensembl Resources; gnomAD, the Genome Aggregation Database; LOVD, Leiden Open Variation Database; PCAWG, Pan-Cancer Analysis of Whole Genomes; TCGA, The Cancer Genome Atlas Program; UCSF, University of California, San Francisco

An overview of all analyses and data integration is provided in Fig. 1.

### Collection, alignment, and phylogenetic analysis of mammalian TREX1 sequences

We employed phylogenetic comparisons to investigate molecular evolution of the TREX1 protein in mammalian species and to analyze conservation of its amino acid residues among species of placental mammals. To achieve this goal, we employed a multi-step phylogenetic approach to identify and analyze full length homologs of the TREX1b isoform (Fig. S2). In the initial stage of data collection, we collected all protein homologs of the canonical human TREX1b (314 aa) protein sequence (NP_338599.1), which was used as query for a sequence similarity search of the curated National Center for Biotechnology Information (NCBI) RefSeq database (Fig. S2). This was performed using blastp (Protein BLAST) similarity search (https://blast.ncbi.nlm.nih.gov/Blast.cgi) [70] with default parameters (*Max target sequences* = *5000; Expect threshold* = *0.05; Automatically adjust parameters for short input sequences; Word size* = *6, Max matches in a query range* = *0; BLOSUM62 matrix; Match/Mismatch Scores* = *1/* *−* *2; Gap costs* = *Existence:11/ Extension:1; Conditional Compositional score matrix adjustment; Low complexity regions filter*). The output of the initial blastp search included 899 RefSeq isoform sequences of the two paralogous proteins TREX1 and TREX2 from a broad range of species (Table S1). They included human TREX2 and human TREX1b and TREX1c isoforms. Five short sequences from vertebrate and invertebrate non-human species with length ≤ 130 aa were not included in the alignment. Information about these sequences is provided Table S1. The resulting dataset contained 894 TREX1-like and TREX2-like proteins from a broad range of species. This dataset was used for initial alignment by the CLUSTALW 2.1 sequence alignment software [71] at GenomeNet at Kyoto University Bioinformatics Center (https://www.genome.jp/tools-bin/clustalw) with the following parameters: *slow/accurate alignment, gap opening penalty* = *15, gap extension penalty* = *0.1, BLOSUM weight matrix*. The resulting alignment was used to infer an initial phylogenetic tree using the MEGA X software [72] by the neighbor-joining (NJ) [73] method, using default parameters (Fig. S2): amino acid substitution model with Poisson correction for multiple hits in distance matrix calculations, substitution rates among lineages following gamma distribution with the gamma (shape) parameter = 1, and homogenous substitution patterns among lineages. Positions containing insertions and deletions (indels, or gaps) were excluded using pairwise deletion in pairs of sequences under comparison.

Since human TREX2, a shorter protein which shares slightly over 40% sequence identify with TREX1, has very distinct molecular and cellular roles from TREX1 [8, 74], we narrowed our subsequent phylogenetic analysis to TREX1 protein sequences (Figs. 1, S2 and S3; Data S1 and S2). The initial phylogenetic tree of 894 TREX1 and TREX2 sequences inferred by MEGA was visually inspected using the Dendroscope tree viewer v. 3.8.8 [75], and the largest monophyletic subtree containing human TREX1 isoforms and other annotated TREX1 sequences but no TREX2 sequences was identified. These TREX1 sequences were extracted for further in-depth analysis of relationships among TREX1 proteins from different species. Since NCBI RefSeq does not contain data on the longest human TREX1a isoform, which is expressed in human glia [28], we added TREX1a to the collection of TREX1 sequences, using the protein sequence AAH23630.1 from the NCBI GenBank GenPept database (https://ncbi.nlm.nih.gov/protein/) (Table S1; Fig. S2). The resulting collection included TREX1 protein sequences from multiple species and included all three human TREX1 isoforms. Human TREX1a (GenBank ID AAH23630.1) has an additional 55 aa N-terminal region prior to the start of TREX1b (RefSeq ID NP_338599.1), whereas human TREX1b has an additional 10 aa N-terminal region prior to the start of TREX1c (RefSeq ID NP_009179.2). This collection of TREX1 sequences was aligned de novo using CLUSTALW 2.1 [71] at GenomeNet at Kyoto University Bioinformatics Center using the same parameters as those for the initial collection of TREX1 and TREX2 sequences. The resulting alignment was manually edited and visualized using JalView multiple sequence alignment workbench v. 2.11.2.6 [76].

Due to the divergence among mammalian TREX1 protein sequences and the existence of multiple TREX1 isoforms, after obtaining the final alignment of 199 TREX1 proteins we subsequently restricted our analysis of TREX1 sequence conservation and phylogenetic inference to full length protein orthologs of the canonical human TREX1b isoform (Figs. 1, S2 and S3; Data S1). Sequences with only partial length homology to human TREX1b or those representing additional isoforms were removed. Notably, the removed sequences with partial homology to human TREX1 included two isoforms from marine mollusk *Mizuhopecten yessoensis* from the scallop family *Pectinidae* (XP_021352012.1 and XP_021352011.1 for uncharacterized protein LOC110449460; Table S1), suggesting the presence of TREX1-like proteins in invertebrates. The remaining final alignment included a collection of 168 TREX1 proteins from mammalian species only, including human TREX1b and its homologs from 167 unique species of mammals. Each mammalian TREX1 sequence was represented by the shortest single isoform with full length homology to human TREX1b. The N- and C-terminal ends of the final mammalian alignment extending beyond the start or the end of the human TREX1b isoform were trimmed.

The alignment of unique 168 mammalian TREX1b protein homologs included 2 sequences of monotremes (egg-laying mammals platypus and echidna) and 7 sequences from marsupials. The inspection of the sequence alignment showed that TREX1b sequences from monotremes, marsupials and placental mammals each contained unique C-terminal domains (Data S1). To identify possible protein or nucleotide sequence homologs of these domains outside monotremes and marsupials, we used NCBI blastp and blastn sequence similarity searches of the unique TREX1b protein and RNA transcript C-terminal sequences of platypus, koala, and human against the nr database of all GenBank sequences (protein and RNA transcript nucleotide sequence GenBank accession numbers: XP_028906636.1 and NC_041749.1, respectively, for platypus, XP_020854878.1 and 020999219.1 for koala, and AAH23630.1 and NM_033629.6 for human TREX1b). An additional blastn search of the GenBank nr database was performed using a 39-nt palindromic AT-rich DNA sequence in the C-terminal domain of the koala *TREX1* gene (Table S2). DNA and RNA structure prediction of that 39-nt palindromic koala sequence was performed using the mfold [77] (http://www.unafold.org/) and RNAstructure [78] servers, respectively.

The 3D structure of the C-terminal protein region of the TREX1 orthologs from *Ornithorhynchus anatinus* (duckbill platypus) and *Phascolarctos cinereus* (koala) was investigated using AlphaFold [79]. Additionally, we examined the potential domains in the C-terminal regions from both species using the protein family and domain classification resource InterPro (https://www.ebi.ac.uk/interpro/) [80] and the deep learning model for transmembrane topology prediction and classification algorithm DeepTMHMM (https://dtu.biolib.com/DeepTMHMM) [81].

Due to the presence of unique C-terminal domains in placental, monotreme and marsupial TREX1b proteins, conservation analysis of TREX1b amino acid residues was restricted to 159 unique TREX1b homologs from different species of placental mammals (Figs. 1, S2 and S3; Data S2). Alignment conservation metrics of 159 TREX1b homologs (Placental consensus, Placental consensus support, Placental quality, Placental occupancy, and Placental conservation [82] ranging from not conserved 0 to highly conserved 11) in placental mammals, were generated by JalView [76] and exported. These metrics and conservation of each TREX1b position in the alignment of 159 placental mammals were integrated in the combined dataset of human TREX1 variants (Fig. 1).

To investigate evolutionary relationships among TREX1b sequences, we inferred separate phylogenetic trees for 168 mammalian sequences and for 159 placental mammals only. The trees for both datasets were inferred using the maximum likelihood (ML) [83] and the neighbor-joining (NJ) [73] methods. Indels were excluded from all analyses in pairs of sequences under comparison, using pairwise deletion. Significance of phylogenetic clustering was evaluated using 1000 bootstrap replications. Phylogenetic tree inference by the NJ method was performed using MEGA X [72] with Poisson correction for multiple hits in distance matrix calculations, using the gamma distribution of substitution rates among amino acid sites, with the gamma parameter = 1 and homogenous substitution patterns among lineages. ML trees were inferred using MEGA 11 [84], assuming variable amino acid substitution rates among sites, with 4 discrete gamma categories under the Jones-Taylor-Thornton model (JTT) method, using site coverage cutoff at 95%, nearest neighbor interchange (NNI) for the ML heuristic method, and automatic inference of the initial tree. Species names and other GenBank descriptors of each sequence were extracted from Table S1 and appended to the trees in the Newick format using our custom software, which was developed using R v. 3.5.3 and 4.2.1. The trees were visualized using MEGA 11 [84].

### Collection and annotation of previously reported human TREX1 sequence variants

We collected available germline and somatic DNA and protein TREX1 sequence variants reported in public databases (Table S3). DNA variants were collected for both coding and noncoding parts of the entire *TREX1* gene. Using available annotations for the TREX1 a, b, and c isoforms, we further annotated those variants which were located within each isoform. To improve the accuracy of data integration, we initially merged the variants from different public datasets at the genomic level and then further integrated those variants that resulted in identical protein changes. The primary focus of our study was on the canonical TREX1b isoform, which has the highest reported levels of expression and activity [18, 24, 27]. Based on the genome and protein positions of the variants and available annotations from individual data sources, we also annotated variants in the TREX1 a and c isoforms and in adjacent noncoding regions.

*COSMIC* COSMIC (Catalogue of Somatic Mutations in Cancer) (https://cancer.sanger.ac.uk/cosmic) is a comprehensive resource on somatic and some germline variants in tumor patient samples, cancer cell lines, and cancer organoids [64]. Its portal provided overlapping variant annotations for the entire *TREX1* gene and for 6 predicted transcript isoforms. Only two of these isoforms, b and c, are included in the curated NCBI RefSeq database (https://ncbi.nlm.nih.gov/refseq/; Table S1). We focused our variant collection and annotation on the *TREX1* transcripts a, b, and c. which were reported to be expressed [18, 24, 27, 28]. We downloaded and merged COSMIC genome variant data for the entire *TREX1* gene (ENST00000629913.3), which includes all three isoforms a, b, and c, and for both functional *TREX1* isoforms: *TREX1b* (ENST00000625293.2) and *TREX1c* (ENST00000444177.1) (Table S4). For each of these entries, we downloaded their table of variants, and separate tables with data on mutation distribution and total unique samples. The data on mutation distribution and total unique samples for each COSMIC entry were merged by the “*CDS Mutation*” field. The three resulting tables for individual entries were subsequently merged into a single integrated data file containing COSMIC information on all samples and all reported variants. Duplicate *TREX1* variants present in multiple COSMIC entries were removed.

*TCGA* Zip data archives for each of the 32 TCGA Pan-Cancer Atlas studies involving 10,967 samples were downloaded from cBioPortal (https://www.cbioportal.org/datasets) [66]. Files containing mutation and GISTIC-derived discrete copy number levels were searched for TREX1 variants. TREX1 mutations were summarized at the genomic and protein level across the 32 Pan-Cancer Atlas studies. Frequencies of TREX1 discrete copy number levels (2 = amplification, 1 = gain, 0 = diploid, -1 = shallow deletion, -2 = deep deletion) were computed for each study (Table S5). Detailed descriptions of TCGA study abbreviations were obtained from the NCI Genome Data Commons (https://gdc.cancer.gov/resources-tcga-users/tcga-code-tables/tcga-study-abbreviations; https://www.cancer.gov/ccg/research/genome-sequencing/tcga/studied-cancers).

*LOVD* LOVD (Leiden Open Variation Database) integrates open source variant data from multiple independent projects [62]. We searched the LOVD^3^ portal (https://www.lovd.nl) for the *TREX1* gene data. The data for NM_033629.3, which represents the functional *TREX1b* isoform and is the only *TREX1* transcript in LOVD^3^ with a current NCBI RefSeq record, were downloaded. All LOVD^3^ annotations of *TREX1* variants were related to germline autoimmune or autoinflammatory disorders.

*ClinVar* ClinVar provides a curated publicly available archive of human genetic variants and interpretations of their significance to disease [61]. All *TREX1* variant annotations in ClinVar were related to germline autoimmune or autoinflammatory disorders. ClinVar data were downloaded from the ClinVar portal (https://www.ncbi.nlm.nih.gov/clinvar) by searching for the *TREX1* gene.

*Decipher* Variants from clinical patient samples from Decipher (DatabasE of genomiC varIation and Phenotype in Humans using Ensembl Resources) [63] were obtained by searching the Decipher portal (https://www.deciphergenomics.org/) for *TREX1* data. The Decipher database contained two clinical variants, both related to germline autoimmune or autoinflammatory disorders.

*gnomAD* We downloaded *TREX1* variant data in human population samples reported in gnomAD (The Genome Aggregation Database) [67] (https://gnomad.broadinstitute.org). We used gnomAD v. 3.1.2, which represents population variants from 76,156 human genomes aligned to GRCh38 [67]. After exporting the gnomAD *TREX1* DNA variants, each variant was annotated based on whether its allele frequency in the entire gnomAD database exceeded 0.0011 (Elevated) or did not exceed that threshold (Low), using the frequency cutoff for *TREX1* pathogenic variants suggested by the VarSome portal (https://varsome.com), which integrates human variant data from multiple sources [85].

*Integration of human TREX1 variant data from different sources* All human *TREX1* sequence variants collected from different databases were initially merged at the DNA level by matching their genome cordinates and specific molecular changes. COSMIC, Decipher, and gnomAD datasets listed DNA variants based on the GRCh38 (hg38) version of genome assembly. LOVD and ClinVar reported the variants using both hg19 and GRCh38 genome assembly versions. For TCGA variant data, only hg19 genome position information was available. Variant data from COSMIC, ClinVar, LOVD, Decipher, and gnomAD were merged using GRCh38 genome positions as a reference. Subsequently, the TCGA *TREX1* DNA variants with exact matches to LOVD or ClinVar data were merged using hg19 genome assembly positions. For the remaining TCGA variants without such matches, we used the lift function from liftOver library in R to convert hg19 to GRCh38 positions, and used those converted positions to integrate them with the full *TREX1* variant dataset.

After merging *TREX1* variants at the genome level, which resulted in 631 unique DNA variants (Table S6), we further integrated them based on available annotation of resulting protein changes. All genomic variants resulting in the same TREX1 protein change were grouped. We manually curated the merged data to ensure that the resulting protein variants (Table S7) were unique and preserved information about underlying DNA changes.

The information from each resource, e.g., the predicted or evidence-based impact or pathogenicity assignment of the variants, was preserved and included separately for each data source (Tables S6 and S7), We curated the merged data based on the chromosomal location and genome positions of the variants. Such filtering removed a small number of entries outside the *TREX1* gene and its adjacent 5ʹ and 3ʹ noncoding regions, e.g., we removed a *SHISA5* gene variant on the *X* chromosome, which was initially downloaded from LOVD^3^ with *TREX1* variants. Additionally, after merging the sequence variants with identical genome positions and amino acid changes, we verified the concordance of their rsIDs among different data sources, if those identifiers were available. We also verified the concordance of cancer variants and samples between TCGA and COSMIC datasets. TCGA *TREX1* sequence variants were also provided as part of COSMIC data, whereas the COSMIC dataset also contained variant data from additional samples. Any sequence variant not passing a quality check was manually curated, and discrepancies were resolved using original biomedical publications.

*Integration of AlphaMissense predictions of pathogenicity of nonsynonymous TREX1 variants* AlphaMissense [68] provided predictions of pathogenicity of all possible nonsynonymous variants in the two functional TREX1 isoforms: TREX1b (ENST00000625293.3, also listed under the Uniprot ID Q9NSU2 as the canonical isoform) and TREX1c (ENST00000444177.1), which are the only isoforms included in NCBI RefSeq (Table S1). We excluded AlphaMissense predictions for several additional predicted short isoforms with no reported biological evidence. We downloaded AlphaMissense predictions from https://zenodo.org/records/8208688 and used SQL to extract predicted pathogenic scores separately for DNA and protein varaints. We extracted the positions and AlphaMissense pathogenicity scores of all substitutions within the TREX1 b (Uniprot ID Q9NSU2) and c (ENST00000444177) isoforms. Predicted AlphaMissense pathogenicity scores were merged separately for protein substitutions (Table S7, with AlphaMissense protein variant data merged based on protein positions and type of protein substitution), and for DNA substititutions (Table S6, with AlphaMissense DNA variant data merged based on GRCh38 genome positions and the type of DNA change).

A heatmap image of AlphaMissense pathogenicity predictions for the canonical TREX1b isoform was obtained from the AlphaFold Protein Structure Database (https://alphafold.ebi.ac.uk/) [79, 86], under the open Creative Commons license CC BY 4.0, for the entry AF-Q9NSU2-F1-v4 (UniProt reference protein sequence Q9NSU2).

### Integration of measures of TREX1 protein sequence conservation in placental mammals

To merge placental mammalian conservation data, we mapped the positions in the alignment of TREX1b protein sequences from 159 placental mammals to the protein positions of the human canonical TREX1b isoform (NCBI RefSeq ID NP_338599.1), accounting for indels in the alignment. Based on the matched positions, we added 5 columns to the list of human TREX1 protein variants from Table S7, which represent summary alignment metrics generated by JalView [76]: Placental consensus, Placental consensus support, Placental quality, Placental occupancy, and Placental conservation [82]. We also integrated placental mammalian alignment and conservation metrics for those human TREX1b protein positions which did not have any reported human variants and therefore were not listed in Table S7. Additionally, based on the alignment of 159 placental mammalian sequences, for each human protein sequence variant in Table S7 we reported all species of placental mammals whose amino acid at that position matched that human variant. The summary information incorporating the human TREX1b protein variants and TREX1b conservation in placental mammals is provided in Table S8.

### Collection of TREX1 copy number variation and structural variation data

*TREX1* copy number alteration (CNA) data in patient cancer samples from TCGA were collected and processed as described above, and integrated with TREX1 sequence variant data (Table S9). Survival analysis of *TREX1* CNA at the single gene level in individual cancer histologies and the comparison of co-occurrence of such events with the loss or gain of the entire 3p arm was performed using cBioPortal (Fig. 1). We also analyzed the co-occurrence of *TREX1* CNA with the loss or gain of the 3q arm, in order to assess how often *TREX1* CNA events occurred as part of the loss or gain of the entire chromosome 3.

Additionally, we examined gene level *TREX1* CNA data in 2802 samples from 38 tumor categories of the Pan-Cancer Analysis of Whole Genomes (PCAWG) dataset [87, 88] using the PCAWG Xena hub (https://dcc.icgc.org/pcawg) of the UCSC Xena Browser (http://xenabrowser.net) [89]. We also examined germline *TREX1* copy number variation data in non-cancerous human samples from individuals without any reported disorders and in patients with developmental delays, which are available from the Database of Genomic Variants v. 107 (http://dgv.tcag.ca) [90]. Large genome variants > 50 bp reported in the NCBI database of human genomic Structural Variation (dbVar) (https://www.ncbi.nlm.nih.gov/dbvar/) were also investigated.

### Use of software

The data were processed and merged using RStudio v. 1.4.17 and R v. 4.1.1, using the packages readr, stringr, tidyr, dplyr, liftOver v. 1.16.0 (for converting positions between hg19 and GRCh38 genome assemblies), and DBI v. 1.1.3 and sqldf v. 0.4.11 (for data extraction from large AlphaMissense files).

### Prediction of the functional impact of TREX1 variants

We evaluated the functional impact of nonsynonymous protein sequence variants in those positions of TREX1b which were reported to be mutated in cancer according to the information from TCGA and COSMIC databases. They included nonsynonymous variants at 12 positions: G23, P29, L44, P48, L87, C99, D101, R164, K175, W210, T236, and G306. We modeled the impact of 20 amino acid substitutions at these positions, which had been reported in patients with cancer or autoimmune disease. These variants are listed in Table S10. They include G23D (cancer), G23S (cancer), G23V(cancer), P29S (cancer and autoimmune disease), L44P (cancer), P48L (autoimmune disease), P48S (cancer and autoimmune disease), L87P (cancer and autoimmune disease), C99R (cancer), C99Y (autoimmune disease), D101H (cancer), D101N (cancer), R164Q (cancer and autoimmune disease), R164P (autoimmune disease), K175N (cancer), W210R (cancer and autoimmune disease), T236A (cancer), T236S (cancer), G306E (cancer), and G306R (cancer and autoimmune disease).

The impact of disease-associated TREX1 variants on protein structure stability was modeled with FoldX [91–93] and the INPS-3D server [94]. Both methods estimate changes in free energy (ΔΔG) from an input protein 3D coordinate file. The structure of the human TREX1 protein was downloaded from the RCSB Protein Data Bank (https://www.rcsb.org; PDB code: 7TQQ, chain E) [95]. FoldX 5.0 was run locally using default parameters. Sidechain repair and energy minimization was performed on the entire structure using the RepairPDB function of the FoldX modeling suite. Then, the BuildModel function was used to introduce individual point mutations. The resulting FoldX output corresponds to the mean ΔΔG of unfolding (i.e., − ΔΔG of folding) over 5 runs in kcal/mol estimated for each amino acid substitution.

INPS-3D (inpsmd.biocomp.unibo.it/inpsSuite/) was run on the online server on 7TQQ chain E and ΔΔG of folding in kcal/mol computed for each amino acid substitution of interest were recorded. We also extracted reported impacts of cancer-associated TREX1 variants on protein structure and function from COSMIC-3D (cancer.sanger.ac.uk/cosmic3d/) [69]. The output of FoldX, INPS-3D, and COSMIC-3D, as well as predicted AlphaMissense pathogenicity scores for disease-associated TREX1 protein-changing variants were integrated into Supplementary Table S11.

To investigate whether any of the disease-associated variants would have a functional impact by hindering TREX1 dimerization or interaction with DNA fragment, we used the Find Clashes/Contacts function of the visualization and analysis suite Chimera version 1.17.3 with default parameters [96, 97]. This analysis identified all direct interactions between TREX1 monomers (7TQQ chain B and chain D), as well as between TREX1 monomer (7TTQ chain B) and complexed DNA fragment (7TQQ chain A).

### Association between TREX1 copy number variation, survival, and 3p/3q status in TCGA

Overall survival and progression-free survival information for TCGA Pan-Cancer Atlas samples was downloaded from cBioPortal (cbioportal.org/datasets) [66]. TREX1 status was stratified into two groups: TREX1 wild type and TREX1 loss (i.e., single copy or deep deletion). The association between TREX1 copy number loss and survival was deemed significant if log-rank test *p*-value < 0.05.

The chromosome arm-level 3p and 3q copy number status (i.e., loss, gain, unchanged) of TCGA Pan-Cancer Atlas samples was obtained from cBioPortal [66]. For each Pan-Cancer Atlas tissue type, the fraction of samples with co-incident TREX1 CNA and 3p or 3q gain or loss was determined.

## Results

### Phylogenetic analysis of TREX1 protein sequences showed the presence of unique C-terminal domains in placental mammals, marsupials, and egg-laying mammals

Consistent with an earlier study of TREX1 and TREX2 evolution [21], our search for TREX1b protein sequence homologs identified both TREX1-like and TREX2-like sequences (Table S1). Their initial CLUSTALW-alignment-based clustering (Fig. S1) showed a distinct monophyletic cluster of mammalian TREX1 sequences (data not shown). Interestingly, based on CLUSTALW-based clustering, the two closest sequences to the mammalian TREX1 cluster were uncharacterized sequences XP_021352012.1 (538 aa) and (XP_021352011.1) from the mollusk *Mizuhopecten yessoensis.* Both mollusk sequences had homology to the N-terminal but not the C-terminal part of the human TREX1b and were not included in the analysis of full-length mammalian sequences.

Alignment of 168 TREX1b mammalian sequences (Data S1) demonstrated that placental mammals, marsupials, and egg-laying mammals had C-terminal domains unique to each of these three phylogenetic groups, corresponding to the 79 aa region at positions 236–314 of human TREX1b, 95 aa of TREX1 of platypus (*Ornithorhynchus anatinus*), a monotreme, and 87 aa of TREX1 of koala (*Phascolarctos cinereus*), a marsupial. Such variability in the C-terminal domains in diverse mammalian taxonomical groups is notable, since the C-terminus of the human TREX1 plays multiple important roles. They include TREX1 localization, anchoring to the ER and perinuclear envelope, and degradation of micronuclear DNA; additionally, this region undergoes post-translational modifications, and patients with SLE and RVCL have highly penetrant mutations in this region [27, 29, 98]. Human TREX1 protein variants lacking the C-terminal TMD domain mislocalize to the nucleus and promote DNA damage by inhibiting homology-directed repair (HDR) [30].

Fig. S4A and B show phylogenetic trees inferred for 168 mammalian TREX1b protein sequence homologs using the ML and the NJ methods, respectively. Both trees showed monophyletic separation of monotreme, marsupial, and placental mammals, with the proper basal position of sequences from egg-laying mammals. However, the accuracy of both trees was likely affected by the presence of unique C-terminal domains in each taxonomic group, resulting in the misclustering of more recently separated phylogenetic groups of placental mammals. For example, while *Atlantogenata* species from the placental mammalian superorders *Afrotheria* (cape golden mole *Chrysochloris asiatica,* Cape elephant shrew *Elephantulus edwardii,* African bush elephant *Loxodonta africana,* aardvark *Orycteropus after,* Florida manatee *Trichechus manatus latirostris,* and lesser hedgehog tenrec *Echinops telfairi*) and *Xentarthra* (Linneus’s two-toed sloth *Choloepus didactylus* and nine-banded armadillo *Dasypus novemcinctus*) formed a monophyletic subtree, however it was clustered within the superorder *Boreoeutheria* of placental mammals. Such position contradicts the early separation between *Atlantogenata* and *Boreoeutheria* during evolution of placental mammals [99].

**Fig. 2:**
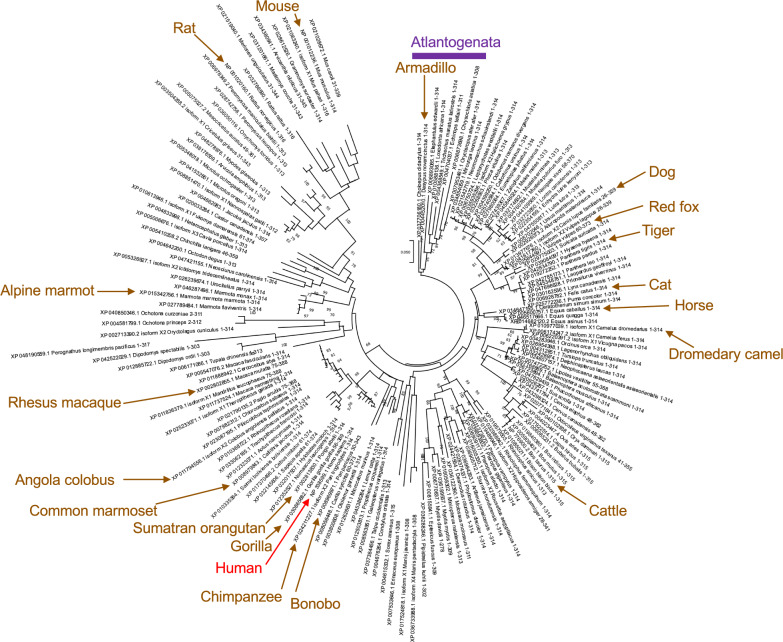
Phylogenetic tree inferred using the maximum likelihood (ML) for 159 TREX1b protein sequences from placental mammalian species. The tree is presented as a circular cladogram and was rooted using *Atlantogenata,* consistent with earlier studies [99]. Species of *Atlantogenata* are indicated by the purple bar. Human TREX1b sequence is shown by the red arrow. Numbers indicate bootstrap support for the tree nodes with support ≥ 60%, out of 1000 bootstrap replications. The scale indicates the number of amino acid substitutions per site

To account for the unique C-terminal domains in egg-laying mammals and marsupials, subsequent analysis of conservation of TREX1b amino acid residues and phylogenetic inference were restricted to 159 unique TREX1b sequences from placental mammals (Figs. 1, S2 and S3; Data S2). Figure 2 shows their phylogenetic tree inferred using the ML method. The NJ tree for the same dataset is presented in Fig. S5. Both trees resulted in a proper separation between *Atlantogenata* and *Boreoeutheria*, although *Atlantogenata* did not have an outgroup position when either tree were rooted at midpoint (data not shown). Consequently, both trees were rooted using *Atlantogenata* as an outgroup, following an earlier taxonomical study of placental mammals using whole genome sequencing data [99].

**Fig. 3:**
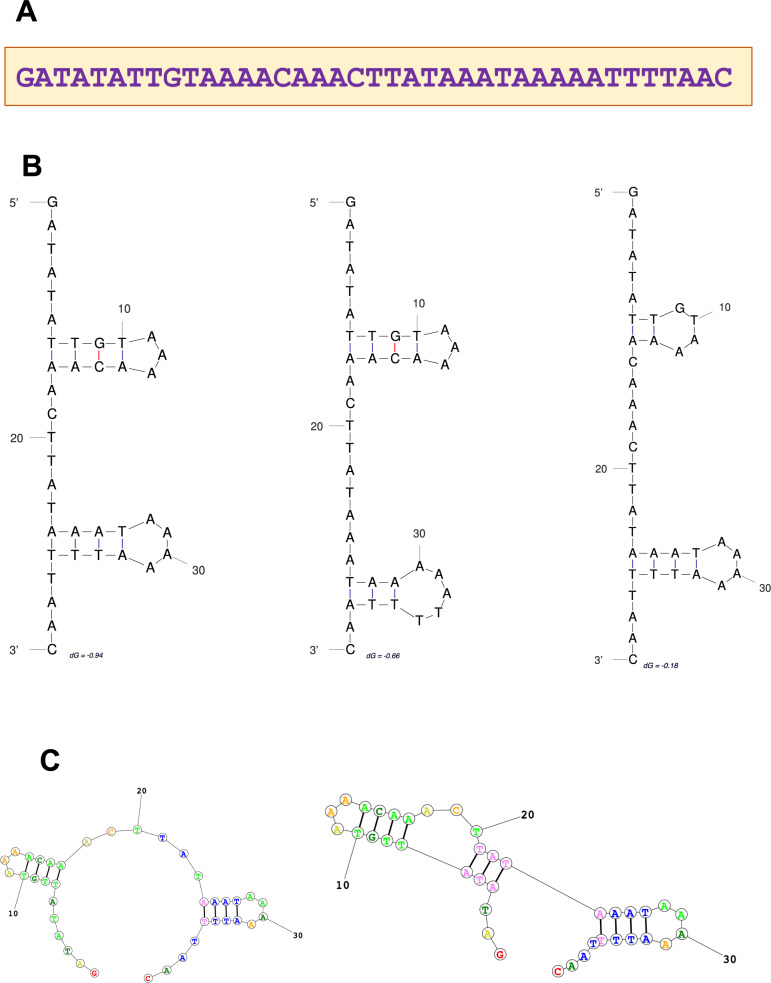
Short 39 nt palindromic DNA sequence in the 3ʹ coding part of koala *TREX1* gene encoding its C-terminal domain. **A** 39 nt koala DNA sequence, which was found to be conserved among marsupials and was also found in invertebrate, plant, and bacterial species (Table S2). **B** Predicted alternate DNA hairpin structures. **C** Predicted alternate RNA hairpin structures

To investigate the origin of unique C-terminal TREX1 domains in different groups of mammals, we searched GenBank for homologs of human, koala, and platypus DNA and protein C-terminal TREX1 sequences. No DNA or protein homologs were found for the human query sequence outside placental mammals, and for the platypus query sequence, none were found outside monotremes. However, the search using koala C-terminal sequence as a query identified a 39-nt palindromic sequence of unknown functionality in the TREX1b C-terminal domain of marsupials (koala, wombat, opossum, possum, and Tasmanian devil), and also in the genomes of invertebrates (insects and mollusks), plants, and bacteria, but not in other vertebrate species. This sequence is highly conserved, with 87–97% identity to the koala query sequence in different species (Table S2), and it contains palindromic repeats, resulting in complex hairpin DNA and RNA structures (Fig. 3), which suggests its possible regulatory role.

To further assess potential significance of the divergent C-terminal protein domains of monotremes and marsupials, we used AlphaFold structure prediction [79] to investigate the 3D structure of the C-terminal region for the TREX1 orthologs from platypus and koala (Fig. S6). In both cases, we observed an elongated disordered region with low per-residue model confidence score. In the case of the koala TREX1 protein, residues 300–320 were predicted to form an α-helix structure. InterPro [80] also predicted that the 95 aa and 87 aa C-terminal stretches, respectively, contained a disordered region followed by a transmembrane region (Fig. S7). Finally, DeepTMHMM [81] also predicted a transmembrane region at the end of the C-terminal tail for the platypus and koala TREX1 proteins, which would be consistent with the predicted α-helix substructure (Fig. S8). The C-terminal region of human TREX1 also contains a transmembrane domain [31]. The presence of C-terminal transmembrane domains in TREX1 of koala and platypus suggests that, similarly to the human C-terminal region, these domains with different sequences may all anchor TREX1 to the ER and perinuclear envelope. Interestingly, using the probabilistic Phobius algorithm [100], InterPro predicted most likely non-cytoplasmic localization for the disordered platypus C-terminal domain and most likely cytoplasmic localization for the koala disordered domain (Fig. S7). Localization of isoforms of the TREX1 ortholog in axolotl, which lacks the C-terminal domain, has been shown to be similar to that of human TREX1 [21]. Future experimental studies may be able to confirm the precise TREX1 localization in monotremes and marsupials and the roles of their divergent C-terminal domains,

### Integration of human TREX1 sequence variants from public datasets

Table S6 summarizes previously reported DNA sequence variants within, upstream, and downstream of the human *TREX1* gene. It also includes protein consequences of each variant in the protein-coding region, clinical significance reported for germline variants, population allele frequencies of variants reported in gnomAD 3.1.2 [67], and AlphaMissense [68] pathogenicity predictions for nonsynonymous substitutions in the TREX1 protein. To date, clinical significance of TREX1 DNA and protein sequence variants has been described only for the germline variants reported in patients with autoimmune and autoinflammatory disorders. This information is provided in Table S6 based on the data from ClinVar, LOVD, and Decipher. In contrast, knowledge about the potential significance of TREX1 variants in cancer is very limited. Table S6 integrates *TREX1* DNA variants reported in cancer patient samples, cell lines, and organoids, based on the data from COSMIC and TCGA.

Additional information from gnomAD included in Table S6 provides data on occurrence of variants, predominantly germline, in human populations. Only 6 *TREX1* DNA variants, corresponding to hg38 positions 48466853 G > A (rs3135943), 48467117 T > C (rs3135944), 48467186 T > C (rs11797), 48467452 A > G (rs55999987), 48467567 G > A (rs3135945), and 48467637 T > C (rs3135946) had elevated (> 0.0011) frequency across human populations in gnomAD. Among them, only 48467452 A > G (rs55999987) resulted in a nonsynonymous protein change, corresponding to E266G in the TREX1b isoform. Interestingly, this variant, which has been reported to have conflicting interpretations of pathogenicity in ClinVar and to be benign or likely benign in LOVD, occurred both in patients with autoimmune disorders and in a mixed adenosquamous carcinoma sample listed in COSMIC (Table S6). Although its frequency (0.00174) across human populations in gnomAD was elevated above the suggested threshold of 0.0011 for *TREX1* pathogenic variants [85]*,* it fell well below the commonly used threshold of 0.01 for rare human variants [101, 102]. The frequency of E266G was also relatively low in individual human populations with data in gnomAD (Table S12), ranging from 0 in East Asian and Middle Eastern populations to 0.00306 in non-Finnish Europeans. In the African/African American population, the low frequency of this variant was 0.00070. These frequencies are similar to estimates from earlier studies, which reported population-specific differences in associations of E266G with disease outcomes. E266G was reported in 5 African patients with SLE carrying this variant, but it was absent in African controls [98]. By contrast, in European populations this variant was present both in controls and in patients with SLE and with early-onset lacunar stroke, and it was not significantly associated with either disease [31, 103]. Consistent with an earlier study [31], we observed low conservation (score = 3) of E at position 266 of TREX1b in placental mammals. E266G was also predicted to be benign or likely benign by AlphaMissense (Table S8). Overall, the evidence for its pathogenicity remains conflicting. It remains to be investigated further whether E266G is a benign variant that occurs in patients with autoimmune and vascular disorders and in tumors due to its presence in the germline, or whether it may modify and increase the risk of disease under certain genetic or environmental conditions.

The noncoding synonymous polymorphism 48467186 T > C (rs11797) at TREX1b protein position 177 had a very high frequency in gnomAD (0.6309), consistent with its assigned benign role. However, due to linkage disequilibrium among different sequence variants, rs11797 was reported to be a representative marker for a risk haplotype, comprised of multiple SNVs in the *ATRIP-TREX1* region*,* which was associated with SLE in European patients [98].

*TREX1* overlaps with an upstream *ATRIP* gene encoding the ATR-interacting protein, and some overlapping genomic variants have been reported to impact certain isoforms of both genes [21, 98]. Our analysis was centered on the impact of TREX1 protein variants, with a special focus on the canonical TREX1b isoform. Table S7 provides an integrated list of TREX1b protein variants compiled from public datasets, additional variants in the C-terminal region of the TREX1a protein isoform, non-coding DNA variants in the 5ʹ and 3ʹ untranslated regions upstream and downstream of the *TREX1* gene, as well as intronic and splice *TREX1* variants. For variants in the 5ʹ region of the *TREX1* gene, their additional impact on *ATRIP* isoforms is listed when such annotation was available from LOVD [62].

Table S8 provides the list of reported human protein sequence variants in the TREX1b isoform and their conservation in placental mammals. While the C-terminal region (human positions 236–314 aa) of TREX1b is unique to placental mammals, many of its protein positions were conserved among placental mammals (Fig. S3; Table S8). Their conservation is consistent with reported associations of a number of variants in that region with human immune and inflammatory disorders [27, 98], as summarized in ClinVar [61] and LOVD [62] (Table S8). Interestingly, AlphaMissense [68] predictions for that region, which were based on variant effects on TREX1b protein structure and conservation in great apes, suggested variants in the TREX1b C-terminal region to be predominantly benign (Fig. S9). As shown in Table S8, all protein substitutions observed in human samples cell lines and organoids for positions 236–314 were predicted by AlphaMissense to be benign, with the only exception of G292D, whose pathogenicity was reported as ambiguous. However, multiple studies [27, 36, 61, 62, 98] reported associations of a number of germline C-terminal variants, e.g. A247P, P290L, and Y305C, with human autoimmune and autoinflammatory disorders, although both ClinVar and LOVD describe all three variants as having uncertain significance or with conflicting reports of pathogenicity. As presented in Table S8 and Fig. S3, these three positions have very high alignment conservation scores (11, representing complete conservation of amino acid residues, or 10, representing conservation of physicochemical properties) among 159 placental mammalians. Not only did A247P, P290L, and Y305C have a low prevalence in human populations based on gnomAD data, but they also did not occur in any other placental mammalian species included in the alignment, providing indirect support for their potential pathogenic effects. In contrast, the C-terminal variants R240S and G306A, which also had been reported in patients with autoimmune and autoinflammatory disorders [27, 36, 61, 62, 98], had conflicting reports of pathogenicity or reports of unknown significance, were present at low frequency in gnomAD (R240S) or were not reported in gnomAD (G306A), and they were not conserved in placental mammals. Their positions had low conservation scores among placental mammals (0 and 4, respectively). These variants occurred in some species of placental mammals (S in the common marmoset *Callithix jacchus* at the protein position corresponding to position 240 of human TREX1b, and A in several fruit bat species, *Pteropus*, at the position corresponding to human position 306).

Outside the C-terminal domain, some variants in the N-terminal catalytic domain, which were reported to be functionally important and to occur in patients with hereditary autoimmune conditions [7], were highly conserved among placental mammals. However, AlphaMissense did not always predict them to be functional. For example, R114H, located in the TREX1 dimerization region [7], results in the loss of TREX1 function in retrotransposon control [1]. It is the most common mutation in patients with AGS, and it also occurs in patients with SLE [8, 27, 36, 98, 104]. This variant is rare in human populations (as indicated by its low frequency in gnomAD), and the physicochemical properties of the R114 position were conserved across placental mammals (conservation score = 10), with a single occurrence of H in Pacific pocket mouse (*Perognathus longimembris pacificus*). Despite multiple reports of the role of R114H in autoimmune response [1, 8, 27, 36, 98, 104], the description of its impact differed among online resources, with Decipher reporting it as pathogenic, LOVD as likely pathogenic or pathogenic, ClinVar reporting conflicting interpretations of pathogenicity, and AlphaMissense predicting it to be benign in TREX1b and likely benign in TREX1c (Table S8). This example underscores the continued need for in-depth evaluation of individual protein variants based on multiple sources of evidence.

For another nonsynonymous variant in the N-terminal region, all data sources highlighted the functional importance of D18N, which is located in the enzymatic DEDDh active-site motif with essential catalytic role [7]. D18N abrogates TREX1 exonuclease function [8, 50] and is an autosomal dominant causative variant for FCL [27, 98]. D18 was highly conserved among placental mammals, with the highest score (11). The D18N variant was absent from gnomAD, consistent with its previously reported minor allele frequency (MAF) of 0 in human populations [98]. It was reported as pathogenic in ClinVar and LOVD and predicted to be pathogenic by AlphaMissense. Similarly to a number of other variants reported as pathogenic or likely pathogenic based on clinical evidence in hereditary autoimmune or autoinflammatory disorders, both R114H and D18N were absent from any cancer specimens in COSMIC or TCGA (Tables S7 and S8).

### TREX1 variants occurring in cancer

**Fig. 4:**
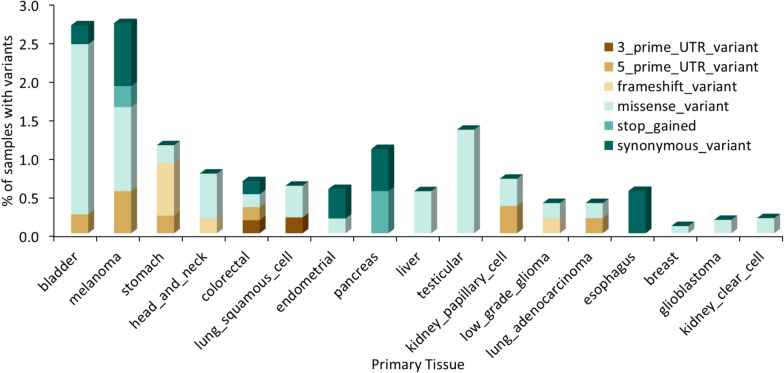
Frequency of TREX1 DNA sequence variants in TCGA. Percent of samples with TREX1 variants in 17 TCGA cancer types. The remaining cancer types had no samples with TREX1 variants. Types of TREX1 variants are indicated with different color stacked bars

Since multiple reports suggested that TREX1 has a protumorigenic effect and contributes to cancer resistance to therapy [13, 15, 29, 43–45, 47–52], the presence of TREX1 variants in tumor samples may have important clinical implications. In TCGA, 16 out of 32 cancer histologies have tumor samples bearing protein-altering TREX1 variants, with bladder cancer and melanoma having the highest frequency of TREX1 variants at 2.7% of tumor samples (Tables S13 and S14; Fig. 4).

While many TREX1 variants with suggested pathogenic effects in germline autoimmune disease were not reported in tumor samples or cancer cell lines, some amino acid substitutions in cancer samples occur at positions that are highly conserved across placental mammals. For instance, G23D, G23S, and G23V were reported, respectively, in stomach adenocarcinoma (STAD) and testicular cancer (TGCT) TCGA samples, and in the COSMIC sample COSS2658231 of adenocarcinoma of large intestine. Glycine (G) at that position has 100% consensus support and the conservation score of 11 among placental mammals (Tables S8 and S10). Similarly, P29S was found in a TCGA bladder cancer sample. This variant was reported in ClinVar to occur in patients with autoimmune disease and to have uncertain significance, however 100% of placental mammals have proline (P) at that position (conservation score = 11; Tables S8 and S10). It remains to be investigated whether these variants may be important for TREX1 enzymatic activity or whether they may occur in patients as benign germline variants.

Some other protein changes were observed in cancer samples at positions that are variable among placental mammals. For example, although C99 is involved in interactions stabilizing human TREX1 dimerization [7], this position is highly variable among mammals and has R (arginine) in the mammalian consensus rather than human cysteine (C), with consensus support of 42.14% and conservation score of 3 for R (Tables S8 and S10). Human variants at this position include the C99Y substitution and a frameshift variant (rs760594164, reported as C99fs and C99Mfs*3) in several patients with autoimmune or autoinflammatory disorders (Table S8). While interpretations of their pathogenicity in ClinVar are listed as uncertain and conflicting, respectively, several reports predicted the germline frameshift variant C99Mfs*3 to be pathogenic in patients with AGS, SLE, and CADASIL [36, 37, 105]. The C99R variant was reported in COSMIC in organoids derived from metastatic samples of patients with gastric adenocarcinoma and adenocarcinoma of the gastroesophageal junction (COSMIC sample identifiers COSS2749229 and COSS2749233; Tables S8 and S10). Both Y (tyrosine) and R at this position are present in many placental mammalian species, with Y found in hog (*Sus scrofa* and *Phacochoerus africanus*) and beaver (*Castor canadensis*) TREX1 sequences, and R occurring in a large number of species (Tables S8 and S10). The presence of the mammalian consensus variant R at position 99 in organoid samples of gastric cancer metastases from different patients is intriguing and may suggest its potential protumorigenic effects.

Human TREX1b position R164, which is involved in DNA binding [7], was also variable among placental mammals (Table S8). The consensus of placental mammals at this position was Q (glutamine) with the support of 46.54% and a low conservation score of 3 (Tables S8 and S10), in agreement with an earlier report by Zhou et al. [7], who noted that R at this position is only present in primate species but not in more divergent mammalian lineages. In humans, R164Q (rs143885312) occurs in patients with autoimmune or autoinflammatory diseases, and also in a bladder cancer sample reported in TCGA and COSMIC, whereas R164P and a premature stop codon at this position were reported in ClinVar in the germline of patients with autoimmune disease (Tables S7, S8, S10, S11, and S14).

All 32 TCGA cancer types in cBioPortal have some samples with loss of one or both copies of *TREX1* (Table S5); frequencies of heterozygous loss range from 0.6 (thymoma) to 86% (kidney clear cell and lung squamous cell carcinomas). We also observed *TREX1* copy gain with lower frequency than instances of copy loss, but also occurring in 31 out of 32 studies (Table S5). Among 10,712 TCGA tumor samples, 3,373 (31.5%) lost one or both copies of the gene*,* 940 (8.8%) gained one or more additional copies, and 6399 samples (59.7%) did not show a change in *TREX1* copy number. Multiple tumors have a combination of a TREX1 protein sequence variant and copy number gain or loss, suggesting that their potential interplay may increase functional impact of TREX1 variants (Table S9).

### TREX1 mutation effect modeling

**Fig. 5:**
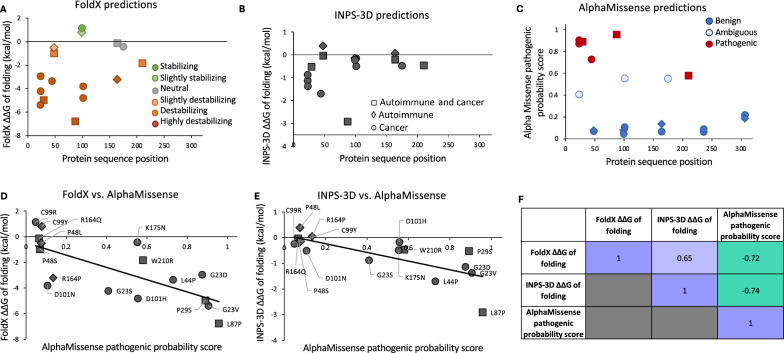
TREX1 mutation effect modeling. **A** FoldX predicted ΔΔG of folding (i.e., − ΔΔG of unfolding) vs. protein sequence position. **B** INPS-3D predicted ΔΔG of folding vs. protein sequence position. **C** AlphaMissense pathogenic probability score vs. protein sequence position. **D** FoldX predicted ΔΔG of folding vs. AlphaMissense pathogenic probability score. **E** INPS-3D predicted ΔΔG of folding vs. AlphaMissense pathogenic probability score. **F** Heatmap of Pearson correlation coefficients between scores from the different prediction methods

**Fig. 6:**
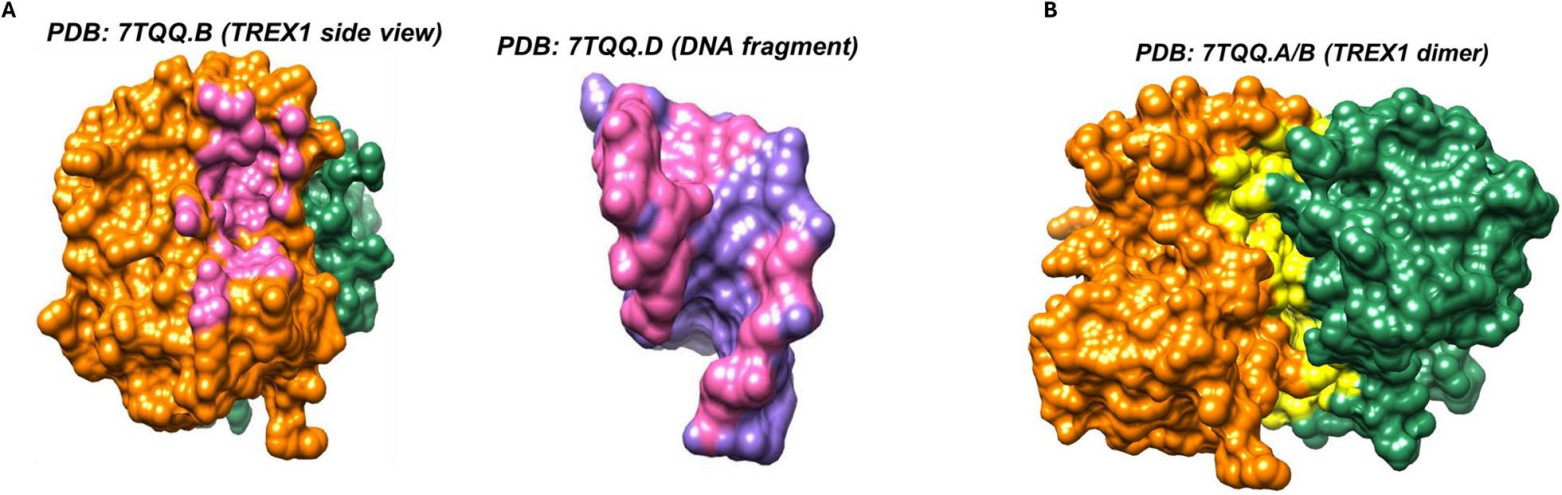
TREX1 structural interactions analysis. **A** Analysis of contacts between TREX1 monomer and DNA fragment. Pink highlights denote sites at the interface of the TREX1 monomer and a complexed DNA molecule determined with UCSF Chimera contact analysis: D18, M19, E20, A21, G23, L24, P25, F26, A80, A81, I84, T85, H124, N125, R128, Y129, I156, K160, R164, S176, Y177, S178, L179, H195, D200. G23 and R164 are known sites of disease-associated variants. **B** Analysis of contacts between TREX1 monomers in a homodimer. Yellow highlights denote sites at the interface between two TREX1 monomers, determined with UCSF Chimera contact analysis: E33, H40, C42, A43, R62, V63, V64, D65, K66, L67, S68, L69, C70, V71, T85, G86, L87, V91, L92, A94, H95, G96, R97, Q98, C99, D101, N103, L104, L107, A110, F111, R113, R114, Q115, P116, H195, T196, E198. L87, C99, and D101 are known sites of disease-associated variants

Since the consequences of human TREX1 protein sequence variants in tumor cells are poorly understood, we modeled the effects of 20 amino acid changes listed in Table S10. These nonsynonymous variants were reported in human samples in any of the 12 TREX1b protein positions that were mutated in cancer. All 20 of these publicly reported human protein variants were in patients with cancer or autoimmune disorders, with no reports of variants in asymptomatic individuals. The results of our modeling analysis of each variant are provided in Table S11. Out of 16 cancer-associated TREX1 variants, 6 (37.5%) were predicted to be pathogenic by AlphaMissense (G23D, G23V, P29S, L44P, L87P, and W210R). Of note, L44P occurred in two hepatocellular carcinoma samples in TCGA (LIHC; Tables S7 and S14).We modeled TREX1 mutations occurring in human tumors to evaluate predicted impact on protein stability and function using structure-based approaches FoldX [91] and INPS-3D [94]. Structures were visualized and residue interactions were analyzed with UCSF Chimera [96]. The changes in Gibbs free energy of folding ΔΔG between WT and mutant predicted by FoldX and INPS-3D both correlated strongly with the AlphaMissense pathogenic probability scores (Pearson correlation coefficient *r* =  − 0.72 and − 0.74, respectively; Fig. 5). Out of the disease-associated variants, those involving G23 and R164 are predicted to disturb the interaction with DNA fragments (Fig. 6), while those variants involving L87, C99, and D101 fall within the region involved in dimerization (Fig. 6). These findings are consistent with those of Zhou et al. who resolved the crystal structure of human TREX1 [7].

### Survival analysis of TCGA patient data

As seen in Table S5, a large proportion of TCGA samples have *TREX1* copy number alterations, with copy number loss being more common. Therefore, we interrogated the association between *TREX1* copy number loss and overall survival (OS) or progression-free survival (PFS) in the TCGA Pan-Cancer Atlas studies. *TREX1* loss was significantly associated (log-rank *p* < 0.05) with worse prognosis in acute myeloid leukemia (OS), bladder urothelial carcinoma (PFS), colorectal cancer (OS and PFS), kidney papillary cell carcinoma (OS and PFS), lower grade glioma (PFS), uterine corpus endometrial carcinoma (OS and PFS), and uveal melanoma (OS and PFS). Kaplan–Meier survival curves are shown in Fig. S10. Due to reported protumorigenic effects of *TREX1*, association of its loss with unfavorable patient outcomes may be influenced by additional factors such as the loss of adjacent genes on chromosome 3.

### Correlation of *TREX1* CNA with chromosome 3 arm-level copy number status

**Fig. 7:**
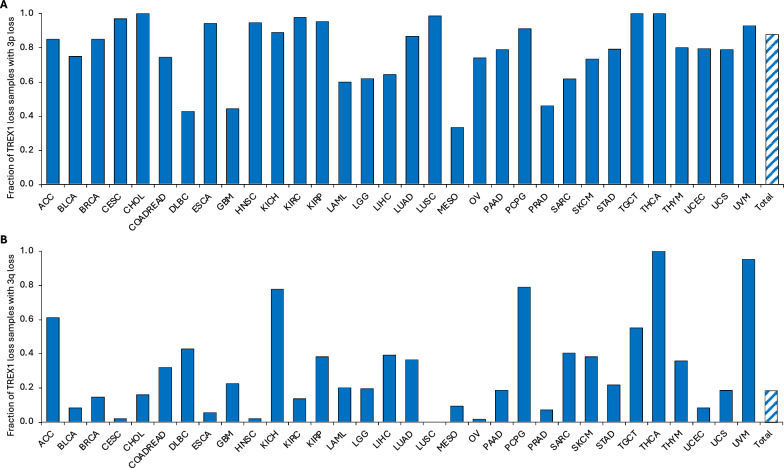
Fraction of samples with co-incident copy number loss in TREX1 and chromosome 3p or 3q in TCGA studies. **A** Samples with TREX1 loss and co-incident 3p loss. **B** Samples with TREX1 loss and co-incident 3q loss. Detailed number of samples per TREX1 status and 3p or 3q status subgroups can be found in Tables S15 and S16

Based on the association of *TREX1* loss with poor cancer patient prognosis, which was observed in our analysis, and previously reported frequent loss of the 3p arm in tumors [54–56, 106–108], we hypothesized that TREX1 copy number alterations in malignant cells may be linked to larger gain or deletion on chromosome 3. Co-deletion of *TREX1* with other genes may have a combined effect on patient outcomes. To test this hypothesis, we computed the fraction of samples with co-incident CNA in TREX1 and in chromosome 3p or 3q arms (Tables S15 and S16). Generally, TREX1 loss occurred with larger loss of the short 3p arm in the majority of TCGA tissue types (Fig. 7A and Table S15). Overall, 88% of samples with *TREX1* loss also had 3p loss. Most frequently, the 3p arm was co-deleted in 85%-100% samples with *TREX1* loss in 19 tumor types including adrenocortical carcinoma (ACC), breast (BRCA), cervical (ESCA), head and neck (HNSC), pancreatic (PAAD), and thyroid (THCA) carcinomas, kidney cancers (KICH, KIRC, and KIRP), non-small cell lung cancer (LUAD and LUSC), pheochromocytoma and paraganglioma (PCPG), testicular germ cell tumors (TGCT) and uveal melanoma (UVM). In contrast, only 18% of samples with TREX1 loss had the loss of the long arm 3q (Fig. 7B and Table S16), suggesting that TREX1 loss tends to occur more specifically with 3p loss. Such event was most common in ACC, KICH, PCPG, TGCT, THCA, and UVM, where 55–100% of samples with *TREX1* deletion also lost the 3q arm, potentially indicating the loss of the entire chromosome 3.

As for *TREX1* gain, a less frequent event than *TREX1* loss, overall 56% of samples with *TREX1* gain also had 3p gain (Fig. S11A and Table S15). *TREX1* gain also co-occurred with 3q gain in about 56% of samples (Fig. S11B and Table S16), suggesting that *TREX1* gain tends to occur within the context of larger chromosome 3 region amplification in specific tumor categories. Most notably, ≥ 70% of the samples with *TREX1* copy number increase in BLCA, CHOL (cholangiocarcinoma), DLBC (diffuse B-cell lymphoma), KICH, KIRP, LAML (acute myeloid leukemia), PAAD, and SKCM (skin cutaneous melanoma) also had 3p gain. Co-occurrence of *TREX1* gain with 3q gain was observed in  ≥ 70% of CESC, CHOL, DLBC, KIRP, LAML, OV (ovarian serous adenocarcinoma), PAAD, and UCS samples, even though some of these tumor categories had very few *TREX1* copy number gain events. For example, CHOL and LAML each had only a single case of *TREX1* copy number gain, which was accompanied by gains of both 3p and 3q arms, i.e., the gain of the entire chromosome 3. These data strongly suggest frequent *TREX1* copy number changes as part of the loss or gain of the 3p arm or the entire chromosome 3 in tumors.

### Structural variation and copy number variation of *TREX1* in a diverse set of tissues

*TREX1* CNA data from non-cancerous germline patient samples in the Database of Genomic Variants v. 107 (http://dgv.tcag.ca) [90] indicated several occurences of *TREX1* copy number loss in patients with developmental disabilities [109], consistent with earlier reports [104]. Interestingly, the Database of Genomic Variants contained data on germline *TREX1* copy number gain in some samples without any reported disorders [110, 111]. This suggests a possibility that some *TREX1* copy number gain may have already existed in the germline of some patients with cancer.

The dbVar database provided information on 139 diverse strutural variants that included copy number changes, insertion of mobile elements, inversions, short tandem repeat variation, tandem duplications, and copy neutral loss of heterozygocity (cnLOH, Table S17). These *TREX1* structural varants were obtained from a variety of source tissues and included both germline variants from healthy individuals [112] and somatic variants (e.g. some of the cancer variants reported in COSMIC [113] and variants from hepatic cysts from patients with polycistic liver disease, or PLD) [114]). Of note, two somatic *TREX1* variants in PLD samples reported in dbVar (nsv2768229 and nsv2768230, Table S17) were a part of a 3p chromosomal region which underwent frequent cnLOH events in patients with PLD [114].

## Discussion

The significance of TREX1 in the regulation of immune response and the effects of its deficiencies on autoimmune disorders have been well documented [2, 3, 7, 8, 27, 35–37, 115]. Recent studies provided new insight into the protumorigenic role of TREX1, its effects on increased cancer resistance to treatment, and suggested TREX1 as an important anticancer drug target [13, 15, 29, 43–45, 47–51, 116]. Underscoring its importance, novel TREX1 inhibitors VB-85680 and VB-86087 have recently been developed as antitumor agents [116]. TREX1 has a protumorigenic effect by inhibiting the cGAS-STING-mediated immune activation within and outside the tumor and by limiting checkpoint activation of the adaptive immune response in the tumor microenvironment [2, 3, 7, 8, 29, 35, 36, 43–45]. Evidence for its protumorigenic role has emerged from the deletion or silencing of the *TREX1* gene. Human *TREX1* was upregulated in HPV-positive cervical cancer cells [117]. Even though gene targeted *Trex1*^−/−^ mice did not have an increase in either mutation rate or incidence of cancer [41], depletion, silencing, or pharmacological inhibition of TREX1 in cancer cell lines or tumor mouse models stimulated the cGAS-STING immune activation, increased the downstream interferon-mediated signaling, and inhibited tumor growth [43–45, 52, 116, 117]. The lack of TREX1 in mouse models also resulted in increased immune activation in the tumor microenvironment, including immune infiltration and activation of CD4+ and CD8+ T cells, and NK cells, prevention of CD8+ T-cell exhaustion, and remodeling of the immunosuppressive myeloid compartment [43–45, 115]. These effects on immune activation in cells where TREX1 was absent or inhibited were conditional on expression of intact components of the cGAS-STING and interferon signaling pathways [43–45, 116]. Furthermore, deletion or inactivating mutations in the C-terminal region of TREX1 in chromosomally unstable cancer cells disrupted micronuclear DNA degradation and increased cGAS activation [29]. STING-independent, DDX3X-mediated cytosolic DNA sensing and immune activation in TREX1-deficient triple negative breast cancer cells has also been reported [115].

Our study provides an important novel resource that includes a collection of TREX1 variants from human tissues and non-human mammalian species. These data include a detailed catalogue of *TREX1* sequence variants, copy number, and structural genome variants, evaluation of the effects of multiple TREX1 protein variants on protein structure stability predictions and on protein molecular interactions, alignment of the canonical TREX1b protein isoform with its orthologs in mammals, and detailed data on conservation of TREX1b protein residues in placental mammals. This information can assist with future targeting of specific TREX1 protein sites in anticancer therapy. It also improves our understanding of germline and somatic *TREX1* variants in human patients. The combined use of a comparative genomics approach and the data on human variants in individuals without reported disorders and in germline and somatic disease samples with structural protein modeling allowed us to evaluate the importance and impact of TREX1 variants and their prevalence in individual cancer categories.

Our comparative analysis provided the surprising discovery that the sequences of the TREX1b C-terminal domains are unique to each group of placental mammals, marsupials, and egg-laying mammals. An earlier study reported the absence of the C-terminal TREX1 TMD domain in axolotl, whose genome contains three isoforms of a putative TREX1 ortholog with functional activity [21]. Similarly, human *TREX2*, from which *TREX1* was suggested to diverge via a gene duplication, lacks the C-terminal TMD region [21]. We also identified two uncharacterized sequences from the mollusk *Mizuhopecten yessoensis*, which were similar to the N-terminal part of the mammalian TREX1. It is therefore likely that the precursor of mammalian TREX1 did not contain a C-terminal region, and that placental mammals, marsupials and monotremes independently acquired different TREX1 C-terminal domains. Given the functional importance of the human C-terminal domain in TREX1 localization and anchoring to the ER and perinuclear envelope, micronuclear DNA degradation, and regulation of the OST complex, and the suggested involvement of mutations or deletion the C-terminal region in human autoimmune and autoinflammatory diseases, OST dysregulation with potential glycosylation defects, mislocalization of TREX1 to the nucleus, and accumulation of DNA damage [2, 7, 16, 27, 29, 30, 32, 33, 98], that acquired region very likely became essential for TREX1 function in placental mammals. Pathogenic variants in the human TREX1b C-terminal region may potentially affect TREX1 interactions with other proteins, and additional studies may be needed to reveal potential pathogenic effects of human C-terminal variants beyond their effect on the protein structure. They may include, e.g., their effects on protein–protein interactions of TREX1 with the ER and the OST complex, and on TREX1 localization, in all of which the C-terminal region plays a critical role [2, 7, 16, 29, 30, 32, 33]. It is notable that the three independent acquisitions of the C-terminal domain by the divergent taxa of mammals resulted in the addition of novel regions, potentially exerting some important influences of the added C-terminal end on overall TREX1 structure. While the origins of the C-terminal regions in each of these three groups of mammals remain unclear, the presence of predicted TMD domains in the C-terminal regions of koala and platypus TREX1 suggests that, similar to human TREX1, they may also be involved in anchoring TREX1 to the ER and perinuclear envelope. The high degree of similarity of the short palindromic sequence within C-terminus of marsupials to invertebrate, plant and bacterial sequences suggests a potential regulatory role of this sequence, which may or may not be related to TREX1 function.

Our study demonstrated that a number of patient tumor specimens carry *TREX1* sequence variants, copy number alterations, or both types of alterations. Notably, many variants with reported pathogenic effects in patients with autoimmune disease (e.g., D18N, R114H, V201D) and many other protein-altering variants which interfere with TREX1 function [8, 10, 27] were not reported in any TCGA or COSMIC cancer samples (Tables S7, S8, S10 and S14). Additionally, a number of variants observed in malignant samples, including G23D, G23S, G23V, D101H, D101N, were not reported in autoimmune disease patients (Table S10). This observation is consistent with the reported protumorigenic role of TREX1 and suggests potential antitumor effects of the variants abrogating TREX1 function. In this case, some functional inactivating TREX1 variants may have opposing effects on different disease phenotypes, promoting the autoimmune disease (if they are present in the germline), but potentially limiting cancer emergence and progression by inhibiting TREX1 suppression of antitumor immunity. However, due to intertwined effects of TREX1 on both the tumor and the immune system [43–45, 115, 116], the role of its variants in cancer may be complex, as some germline *TREX1* variants have been associated with hematological malignancies in patients with autoimmune diseases [46]. For example, compound heterozygous nonsynonymous TREX1 mutations were found in a patient with AGS who developed Hodgkin lymphoma, and decreased prevalence of the minor allele A of the nonsynonymous variant rs11797 (at TREX1b protein position Y177, Tables S7 and S8) was reported in patients with co-occurrence of the autoimmune disease primary Sjogren’s syndrome and MALT (the mainly mucosa associated lymphoid tissue lymphoma type), a type of non-Hodgkin lymphoma [118, 119].

Prevalence of different *TREX1* sequence and copy number variants was variable among TCGA and PCAWG samples from different tumor histologies (Figs. 4 and S12; Tables S13, S15, and S16), suggesting potential tumor-specific differences in the impact of *TREX1* alterations. Of note, two hepatocellular carcinoma samples in TCGA carried the L44P TREX1b protein sequence variant, while organoids from two gastric adenocarcinoma patients carried the C99R variant (Tables S7, S8, S10, and S14), suggesting a possibility of associations of specific TREX1 variants with specific tumor histologies. Additionally, three different nonsynonymous substitutions at position 23 were reported in TCGA and COSMIC for different tumor categories, suggesting possible protumorigenic effects of certain TREX1 changes.

Our modeling predictions for protein positions which had amino acid substitutions in cancer specimens (Table S11) were mostly in agreement with data from other groups. By using comparative analysis of crystal structures of human and mouse TREX1 bound to DNA, Zhou et al. showed that human R164 is directly involved in binding to the 5ʹ end of DNA, whereas mouse TREX1 has Q at that position, which is not involved in DNA binding [7]. Their findings indirectly support the potential impact of the human R164Q variant on disturbing DNA binding. Further experimental validation may be beneficial to evaluate the impact of the human R164Q substitution, which is currently classified in ClinVar as having uncertain significance, due to insufficient evidence (Tables S7 and S8). Crystal structure analyses by Zhou et al. of the *apo* and DNA-bound TREX1 conformations also found the human C99 and K175 positions to be involved in TREX1 protein stability [7]. Their experimental results support our modeling predictions for substitutions at C99. By contrast, the impact of K175N, which was present in bladder cancer and melanoma samples, was less definitive in our predictive analysis (Tables S7, S8, and S11). K175N had been reported earlier in patients with early-onset lacunar stroke, however it was not associated with the risk of that disease [103]. A separate, unpublished study by Hemphill et al. [120] used molecular dynamic simulations to identify G23 as a part of a short stretch that can participate in the TREX1 contact with the 3ʹ end of dsDNA and ssDNA. Their results support our prediction of a potential impact of substitutions at position 23 (G23D, G23V, and possibly G23S) on disruption of TREX1 contact with DNA. The effects of these and other variants on biological activity of TREX1 in malignant and non-cancerous cells require further biochemical validation.

Some cancer variants listed in Table S10 occur at positions such as Y305 and G306, where a single mutated allele is known to be sufficient to cause SLE. Other mutations in cancer samples were at positions C99, R164, and W210, which follow the recessive mode of germline inheritance of AGS, where the presence of two mutated copies is needed for disease manifestation [8, 27]. It is possible that recessive *TREX1* variants may be present in the germline of some cancer patients or emerge as de novo somatic mutations in tumors. In the absence of the second mutated allele, such recessive variant would not affect the TREX1 function. However, TCGA samples from different tumor histologies frequently lost one (shallow deletion), or, less frequently, both copies (deep deletion) of *TREX1*, or gained one (CNA gain) or more (amplification) additional gene copies. PCAWG data confirmed *TREX1* copy number changes in many tumor samples from a variety of histologies (Fig. S12). PCAWG tumor samples frequently had *TREX1* copy number loss (predominantly a loss of one copy) or a gain from 1 to up to 7 additional copies. Many TCGA tumor samples with protein-changing *TREX1* sequence variants had co-occurring *TREX1* copy number alterations (Tables S5 and S9). Given protumorigenic effects of TREX1 via its inhibition of interferon signaling and the promotion of tumor escape from immune surveillance, copy number gain of the normal *TREX1* allele could potentially occur in some tumors as compensatory mechanism if the second somatic or germline allele is mutated. Amplifications of intact *TREX1* may also provide a selective advantage to tumors and have a protumorigenic effect. However, despite the potential advantage of *TREX1* amplification, the number of tumor samples in TCGA with *TREX1* loss was 3.6 times higher than the number of samples with *TREX1* copy number gain (Table S5).

Loss of one or both copies of *TREX1* frequently involved a broader deletion containing the chromosomal region surrounding *TREX1* or the entire short arm of chromosome 3, which are common in many cancer types [54–56, 106–108]. In agreement with previous reports of frequent losses of these chromosomal regions, 88% of the TCGA tumor samples with *TREX1* deletion also lost the entire short arm of chromosome 3. Co-deletion of *TREX1* and 3p was present in ≥ 60% of all cases of *TREX1* loss in 28 out of 32 TCGA tumor categories (Table S15, Fig. 7A). Frequent concurrent loss of *TREX1* with adjacent genes provides the likely explanation for the poor prognosis of patients carrying a *TREX1* deletion in LAML, BLCA, COADREAD, KIRP, GBM, UCEC, and UVM tumors (Fig. S10) and is consistent with previous reports of poor survival outcomes in patients with uveal melanoma who lost the chromosomal region containing the *BAP1* gene at 3p21.1 [57, 121, 122]. However, the results of our survival analysis should be interpreted with caution, as they did not account for patient treatment, tumor grading, or additional patient data that could affect treatment outcomes.

Co-occurrence of *TREX1* protein-changing sequence variants with copy number alterations could abrogate TREX1 function, produce a homodimer protein composed of two mutated copies, or lead to TREX1 protein dimerization abnormalities. If the second functional allele were lost in a tumor, *TREX1* sequence variants that follow a recessive pattern of inheritance in the germline may potentially have tumor inhibiting roles [29]. Such combinations of TREX1 protein-changing sequence variants and copy number loss, or the loss of both copies of *TREX1* may sensitize tumors to DNA damaging therapies. Therefore, in addition to the previously reported associations of low *TREX1* expression and increased DNA methylation of *TREX1* promoter with increased tumor sensitivity to chemotherapy and radiation therapy [47–49, 51, 52], mutations and copy number changes of *TREX1* are likely to play additional contributing roles in tumor response to treatment. Any combination of low *TREX1* expression due to epigenetic inactivation, damaging protein sequence mutations, and *TREX1* copy number loss may create therapeutic vulnerabilities which may improve treatment outcomes. Consistent with this suggestion, cells carrying TMD-deficient TREX1 frameshift RCVL variants were sensitive to genotoxic agents and ionizing radiation, and this sensitivity was influenced by the expression levels of the mutant TREX1 alleles [30]. Based on the extent of the loss of TREX1 function, some of these therapeutic vulnerabilities may be more prevalent in specific cancer categories [51].

Based on the data in the Database of Genomic Variants and dbVar, some copy number changes and structural variants of *TREX1* are present not only in tumors, but also in the germline or non-cancerous somatic tissues (e.g., in the germline of individuals with and without intellectural disabilities and in hepatic cysts of patients with PLD). Since somatic *TREX1* variants nsv2768229 and nsv2768230 in PLD samples are located in a 3p region with frequent cnLOH events in PLD [114], *TREX1* cnLOH events merit further studies to explore their frequency in other conditions and in healthy individuals, and to better understand their possible effects on immune activation. Additionally, many structural variants, including both cnLOH variants nsv2768229 and nsv2768230 in PLD, and many copy number alterations of *TREX1* in cancer also involve, e.g., *RASSF1* (*RASSF1A*)*, TUSC2* (*FUS1*)*, NPRL2, CYB561D2* (*101F6*) at 3p21.3, *BAP1* at 3p21.1, and other important genes at 3p (Table S17), which may contribute to the molecular impact of such events [54–57, 114, 121].

The scale of the currently available sequencing data presents challenges in interpretation of pathogenicity and clinical impact of individual genome and protein sequence variants. There is ongoing development of many novel resources and computational tools to assist with interpretations of individual variants. We performed an in silico characterization of variation in the biologically and clinically important protein TREX1, whose variants may have a potentially widely ranging impact on different human disease outcomes. Whereas some germline TREX1 variants may have pathogenic effects in autoimmune disorders, some of the same functional variants may provide a therapeutic vulnerability in cancer treatment by dampening or abrogating TREX1 activity and increasing antitumor immune response. Our study integrates available data on TREX1 variant activity, providing a novel resource for future in-depth clinical and biological investigation of this protein in a variety of human diseases. Such future investigations may potentially expand the range of diseases beyond autoimmune diseases and cancer, potentially including additional disorders with the late onset and complex contributing genetic and environmental factors. For example, *TREX1* cnLOH variants were present in hepatic cysts of patients with PLD (Table S17) [114]. Rare *TREX1* sequence variants were also reported in patients with early-onset lacunar stroke, although they were not associated with disease risk [103]. Based on the importance of TREX1 in the control of immune activation, and the role of inflammation and interferon response in neurological, neurodegenerative, and musculoskeletal disorders and in clinical treatment [19, 20, 123–125], future studies may investigate whether TREX1 variants may play contributing or modifying roles in a broad range of diseases that involve inflammatory processes.

## Conclusions

We developed a catalog of germline and somatic TREX1 variants in human disease samples, cancer cell lines and organoids, and population-based samples. Placental mammals, marsupials and egg-laying mammals have unique sequences at the C-terminal region of the TREX1b protein, suggesting unique adaptations in each taxonomical group. Functional impact of human cancer-associated TREX1 protein sequence variants was predicted using different approaches with consistent results. While TREX1 protein sequence variants and copy number alterations are present in many cancer histologies, their impact on malignancy may differ from functional effects in autoimmune disorders.

## Supplementary Information

Additional file 1. Data S1. Protein sequence alignment of 168 mammalian TREX1b sequences in Clustal format.

Additional file 2. Data S2. Protein sequence alignment of 159 TREX1b sequences from placental mammals, in Clustal format.

Additional file 3. Supplementary figures.

Additional file 4. Table S1. TREX1 and TREX2 sequences collected using blastp searches of the NCBI RefSeq database using human TREX1b (NP_338599.1) as a query, with addition of human TREX1a isoform from GenPept.

Additional file 5. Table S2. Nucleotide sequence matches identified using blastn searches of the NCBI GenBank nr database using 39-nt palindromic sequence in the C-terminal region of the koala *TREX1* gene as a query.

Additional file 6. Table S3. Public online resources used for collection of human TREX1 genomic and protein sequence variants in human samples, cell lines, and organoids and for annotation of their impact.

Additional file 7. Table S4. Information about COSMIC *TREX1* gene and isoform entries used for collection of *TREX1* variants.

Additional file 8. Table S5. Frequencies of *TREX1 *copy number alterations in TCGA studies.

Additional file 9. Table S6. Information about genomic *TREX1* sequence variants compiled from public databases.

Additional file 10. Table S7. Human TREX1b protein sequence variants reported in public databases, and genomic variants upstream and downstream of the *TREX1b* isoform.

Additional file 11. Table S8. Reported human protein sequence variants in the TREX1b isoform with added data on conservation of each TREX1b position in 159 placental mammalian species.

Additional file 12. Table S9. List of *TREX1* DNA sequence variants with copy number alteration information in TCGA samples.

Additional file 13. Table S10. TREX1b protein positions which were included in the modeling analysis.

Additional file 14. Table S11. TREX1 protein sequence variant modeling results.

Additional file 15. Table S12. Frequency of the E266G (48467452 A>G) variant in different human populations in gnomAD.

Additional file 16. Table S13. Distribution of different nucleotide sequence variant categories in TCGA cancer histologies.

Additional file 17. Table S14. Protein-level TCGA variants.

Additional file 18. Table S15. Copy number status of *TREX1* and chromosome 3p in 32 TCGA cancer histologies.

Additional file 19. Table S16. Copy number status of *TREX1* and chromosome 3q in 32 TCGA cancer histologies.

Additional file 20. Table S17. Human structural *TREX1* variants listed in the dbVar database.

## Data Availability

All data analyzed during the current study are publicly available. TREX1 variant data were collected from public resources listed in Table S3. TREX1 and TREX2 protein sequences are available from GenBank at NCBI (https://www.ncbi.nlm.nih.gov/genbank/), with sequence identifiers provided in Table S1. All data generated during this study are included in this published article and its supplementary information files.

## Abbreviations

aa: Amino acid
ACC: Adrenocortical carcinoma
AGS: Aicardi–Goutières syndrome
BLCA: Bladder urothelial carcinoma
CADASIL: Cerebral autosomal dominant arteriopathy with subcortical infarcts and leukoencephalopathy
BRCA: Breast invasive carcinoma
CESC: Cervical squamous cell carcinoma and endocervical adenocarcinoma
CHOL: Cholangiocarcinoma
CNA: Copy number alteration
cnLOH: Copy neutral loss of heterozygocity
COADREAD: Colon adenocarcinoma and rectal adenocarcinoma
COSMIC: The Catalogue of Somatic Mutations in Cancer
dbVar: Database of human genomic structural variation
Decipher: DatabasE of genomiC varIation and Phenotype in Humans using Ensembl Resources
DLBC: Lymphoid neoplasm diffuse large B cell lymphoma
dsDNA: Double-stranded DNA
ESCA: Esophageal carcinoma
ER: Endoplasmic reticulum
FCL: Familial chilblain lupus
GBM: Glioblastoma multiforme
gnomAD: The genome aggregation database
HNSC: Head and neck squamous cell carcinoma
JTT: Jones–Taylor–Thornton model
KICH: Kidney chromophobe
KIRC: Kidney renal clear cell carcinoma
KIRP: Kidney renal papillary cell carcinoma
LAML: Acute myeloid leukemia
LIHC: Liver hepatocellular carcinoma
LGG: Brain lower grade glioma
LOVD: Leiden open variation database
LUAD: Lung adenocarcinoma
LUSC: Lung squamous cell carcinoma
MALT: Mainly mucosa associated lymphoid tissue lymphoma type
MESO: Mesothelioma
ML: Maximum likelihood
NCBI: National Center for Biotechnology Information
NJ: Neighbor-joining
NK cells: Natural killer cells
NNI: Nearest neighbor interchange
OS: Overall survival
OST: Oligosaccharyltransferase
OVCA: Ovarian serous adenocarcinoma
PAAD: Pancreatic adenocarcinoma
PCAWG: Pan-cancer analysis of whole genomes
PCPG: Pheochromocytoma and paraganglioma
PFS: Progression-free survival
PLD: Polycistic liver disease
PRAD: Prostate adenocarcinoma
RCVL: Retinal vasculopathy with cerebral leukodystrophy
SARC: Sarcoma
SKCM: Skin cutaneous melanoma
SLE: Systemic lupus erythematosus
SNV: Single nucleotide variant
ssDNA: Single-stranded DNA
STAD: Stomach adenocarcinoma
TCGA: The Cancer Genome Atlas Program
TCGT: Testicular germ cell tumors
THCA: Thyroid carcinoma
THYM: Thymoma
TMD: Transmembrane domain
TREX1: Three-prime repair exonuclease 1
UCEC: Uterine corpus endometrial carcinoma
UCS: Uterine carcinosarcoma
UVM: Uveal melanoma
