# Supplementary material for: Prevalence and impact of molecular variation in the three-prime repair exonuclease 1 TREX1 and its implications for oncology
Source: Hum Genomics. 2025 Jun 28;19:73. doi: 10.1186/s40246-025-00785-y (PMC12206365; doi:10.1186/s40246-025-00785-y)
Supplement: Supplementary file 3 — Additional file 3. Supplementary figures. [file 40246_2025_785_MOESM3_ESM.pdf]

Supplementary Figures

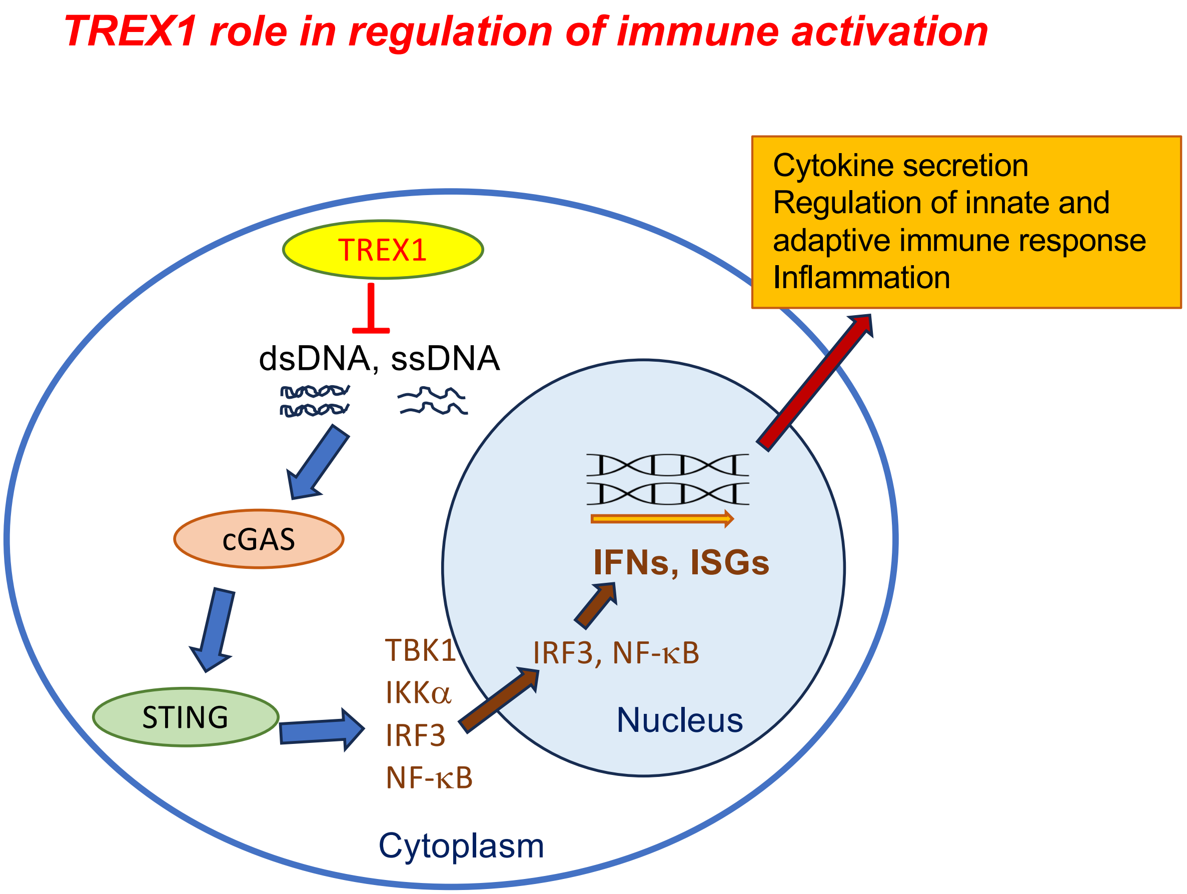

Fig. S1. Role of TREX1 in cytosolic DNA degradation and regulation of innate immune response. The figure summarizes information from previously published original reports and review articles [2-4,15,20,21,125].

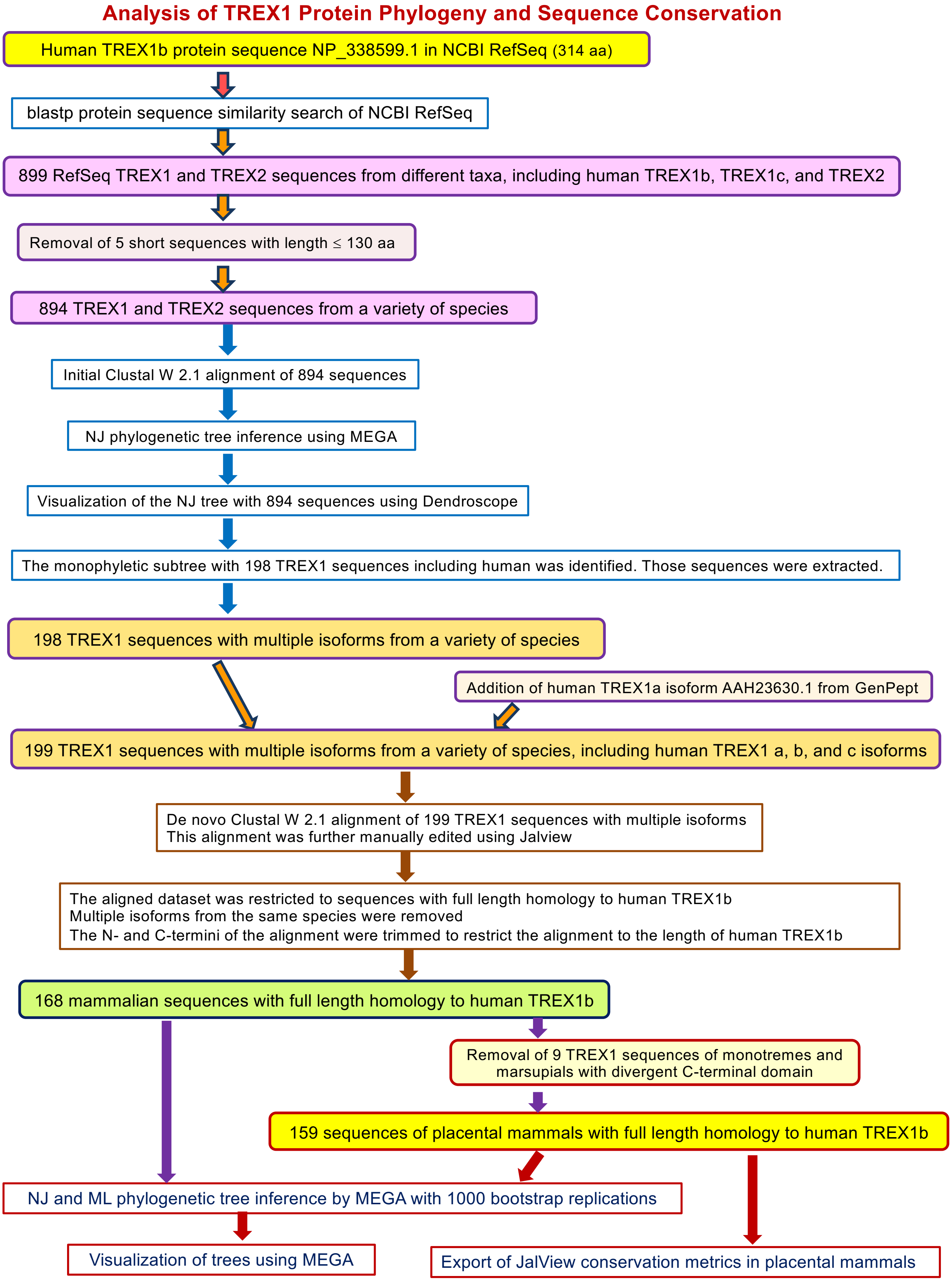

Fig. S2. A workflow of collection, alignment, phylogenetic analysis, and analysis of sequence conservation of mammalian TREX1b homologs. Detailed information about individual steps is provided in the Methods.

Fig. S3 (*next page*). Protein sequence alignment of 159 TREX1 sequences including human TREX1b and its orthologs in placental mammals, exported from JalView. The histogram on the bottom provides alignment conservation metrics (conservation, quality, consensus, and occupancy) in placental mammals, which were generated by JalView and integrated in the combined dataset of human TREX1 variants (Table S8). This alignment was derived from the protein sequence alignment of 168 mammalian TREX1b sequences (Data S1), after removing the 7 marsupial sequences and 2 sequences of monotremes, and removing the gaps unique to those divergent groups. This alignment is provided in Clustal format in Data S2.

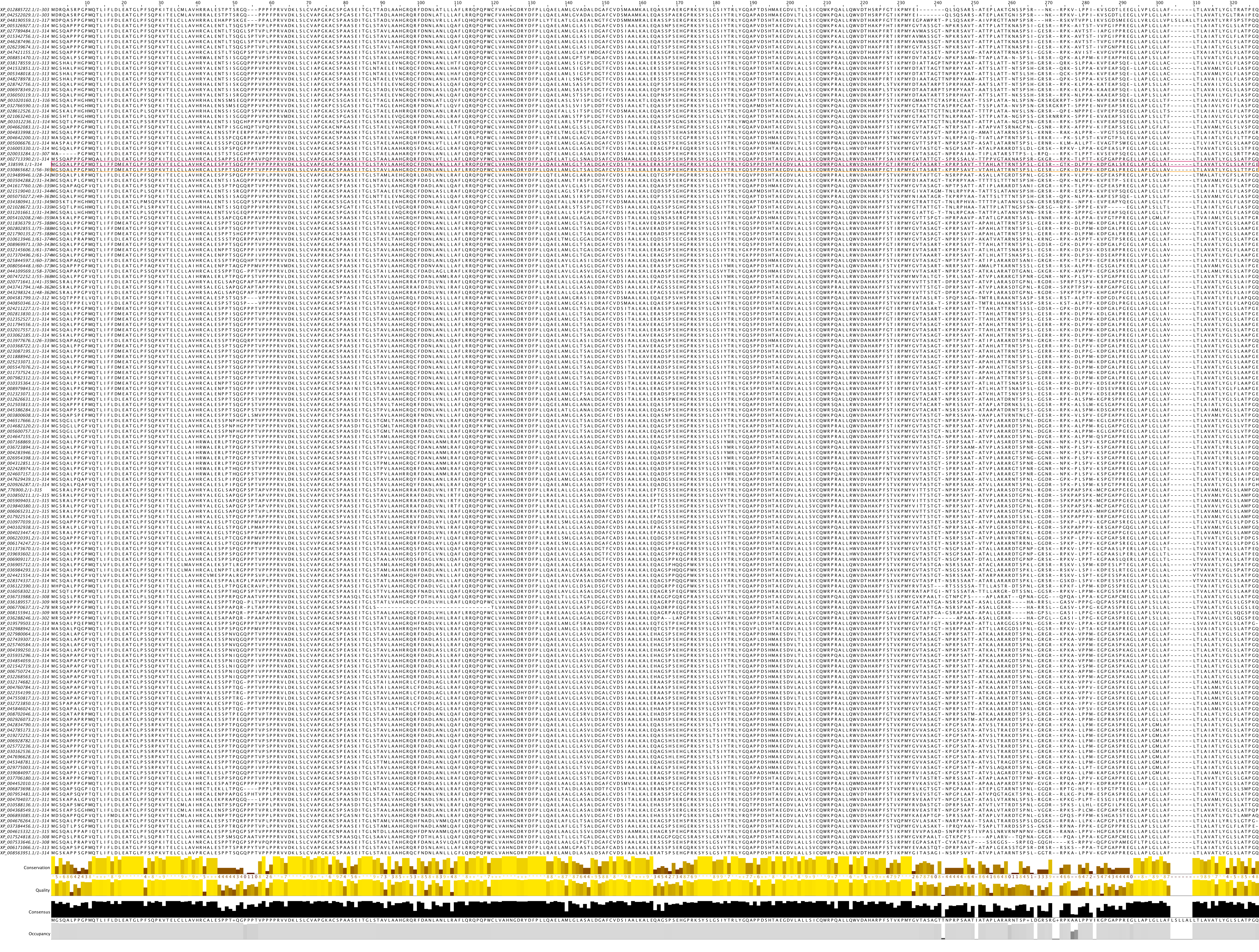
Conservation
Quality
Consensus
Occupancy

Fig. S4 (*following pages*). Phylogenetic clustering of 168 mammalian TREX1b protein sequences. Each tree is presented as a circular cladogram and was rooted at midpoint. Numbers indicate bootstrap support for the tree nodes with support ≥ 60% out of 1000 bootstrap replications. The scale indicates the number of amino acid substitutions per site. Species of monotremes and marsupials are indicated by yellow and blue bars, respectively. Human TREX1b sequence is shown by red arrow. The most recent common ancestor of *Atlantogenata* is marked by a purple circle. **(A)** Phylogenetic tree inferred using the maximum likelihood (ML) method. **(B)** Phylogenetic tree inferred using the neighbor-joining (NJ) method.

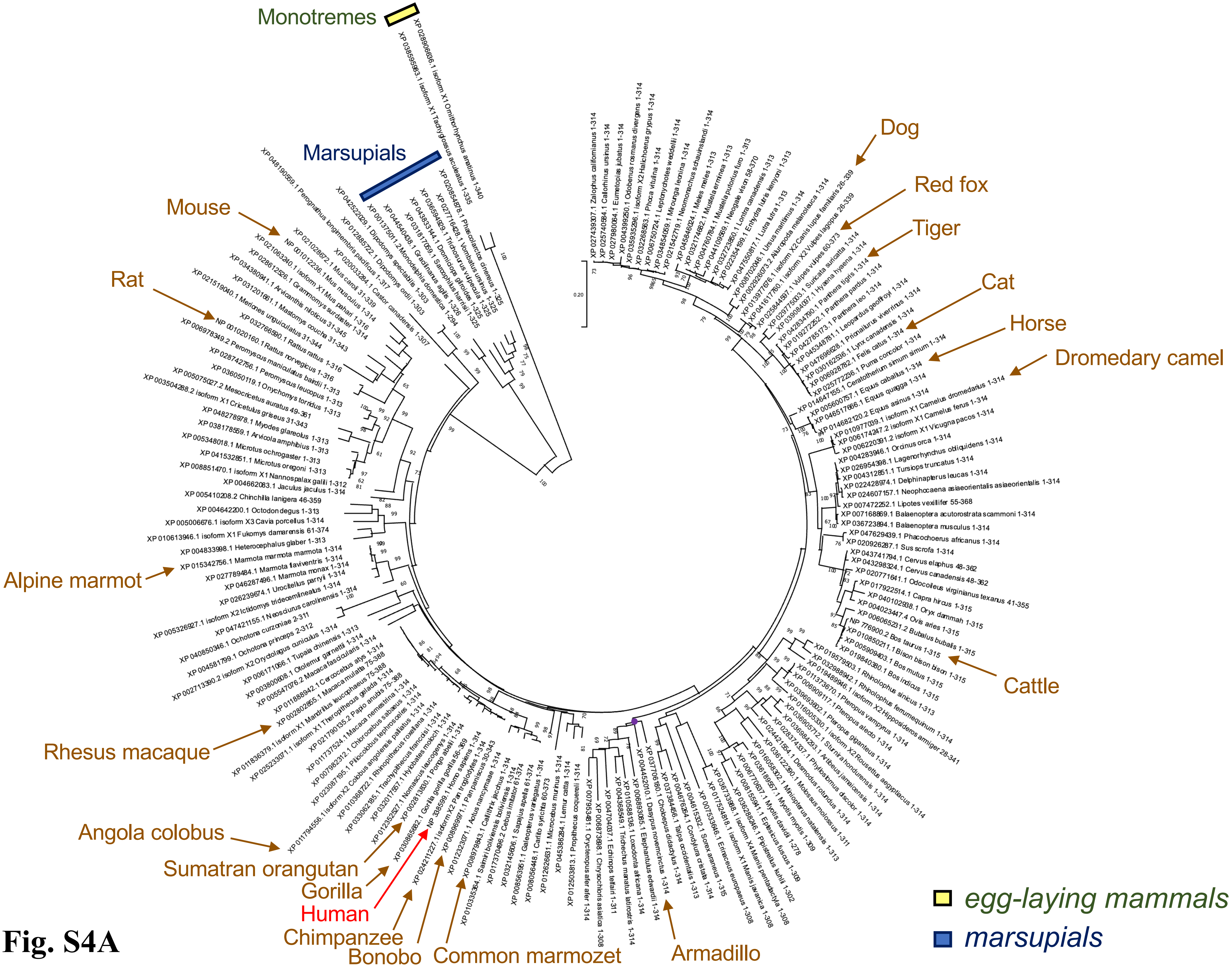

Monotremes
Marsupials
Mouse
Rat
Alpine marmot
Rhesus macaque
Angola colobus
Sumatran orangutan
Gorilla
Human
Chimpanzee
Bonobo
Common marmozet
Armadillo
Cattle
Dog
Red fox
Tiger
Cat
Horse
Dromedary camel
egg-laying mammals
marsupials

Fig. S4A

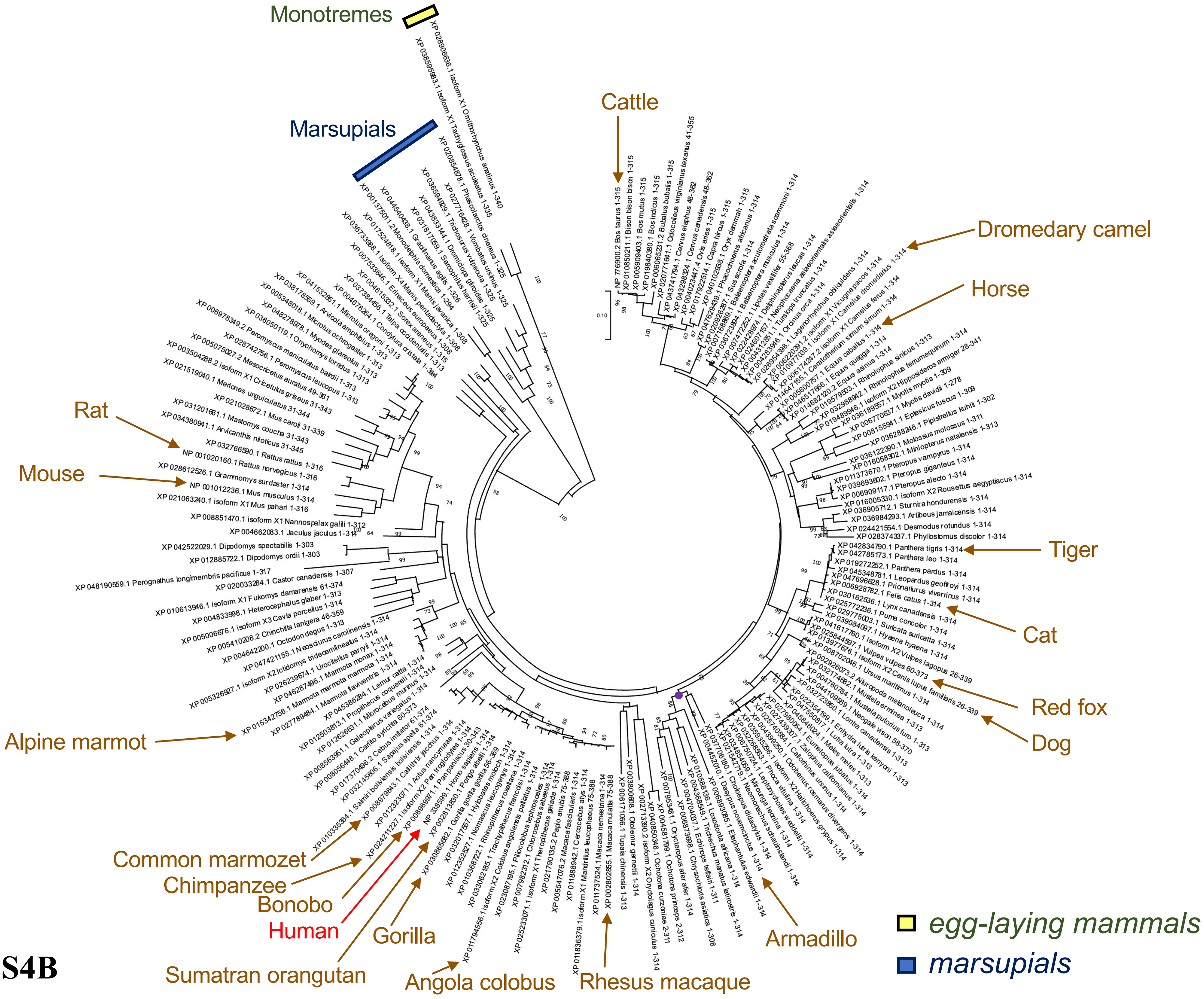
Monotremes
Marsupials
Cattle
Dromedary camel
Horse
Tiger
Cat
Red fox
Dog
Armadillo
Rhesus macaque
Angola colobus
Sumatran orangutan
Gorilla
Human
Bonobo
Chimpanzee
Common marmozet
Alpine marmot
Mouse
Rat
egg-laying mammals
marsupials

Fig. S4B

Fig. S5 (*next page*)**.** Phylogenetic tree inferred using the neighbor-joining (NJ) method for 159 TREX1b protein sequences from placental mammals. The tree is presented as a circular cladogram and was rooted using *Atlantogenata,* consistent with earlier studies [99]. The scale indicates the number of amino acid substitutions per site. Human TREX1b sequence is shown by the red arrow. Numbers indicate bootstrap support for the tree nodes with support ≥ 60% out of 1000 bootstrap replications.

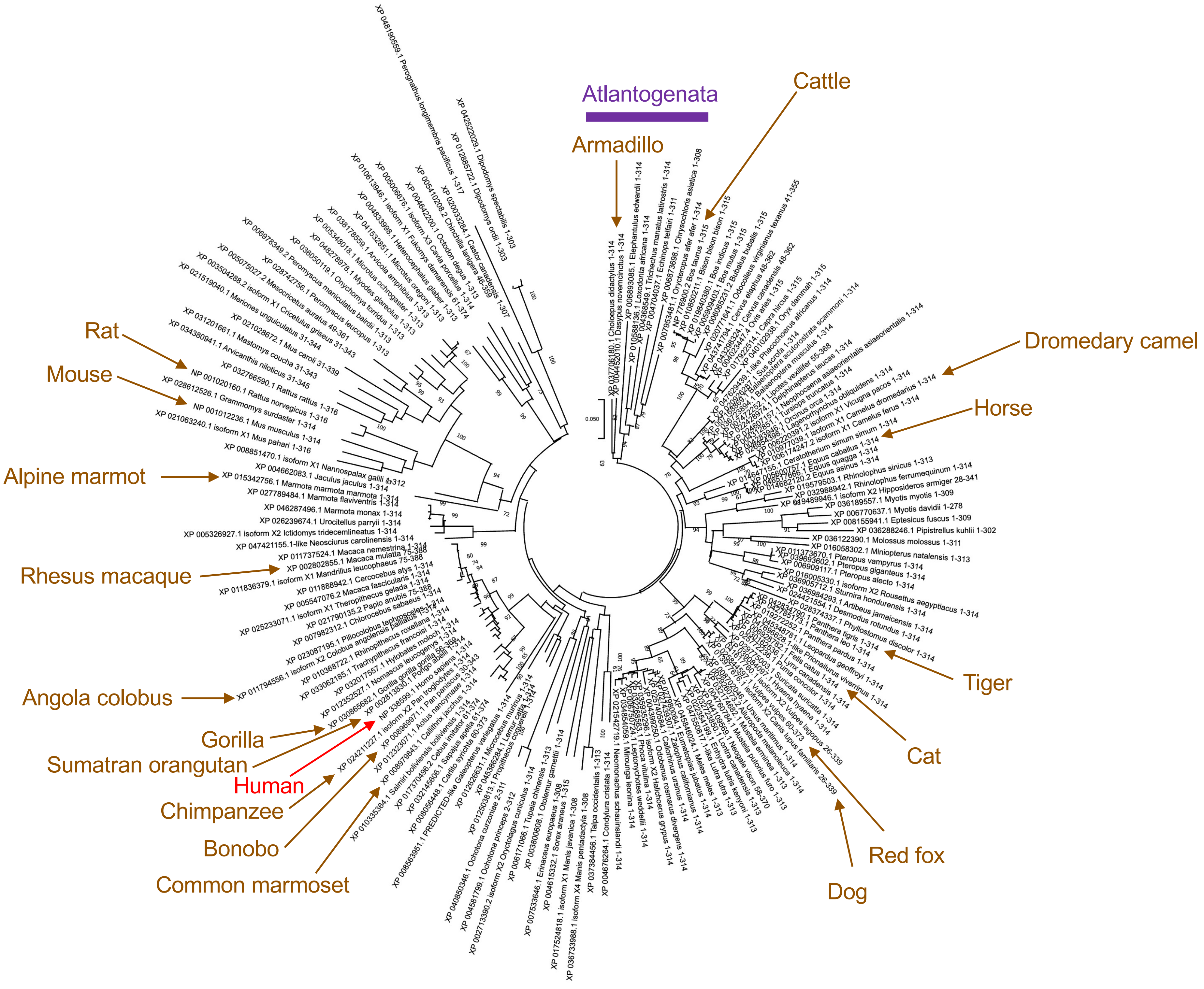

Atlantogenata
Armadillo
Cattle
Dromedary camel
Horse
Tiger
Cat
Red fox
Dog
Rat
Mouse
Alpine marmot
Rhesus macaque
Angola colobus
Gorilla
Sumatran orangutan
Human
Chimpanzee
Bonobo
Common marmoset
0.050

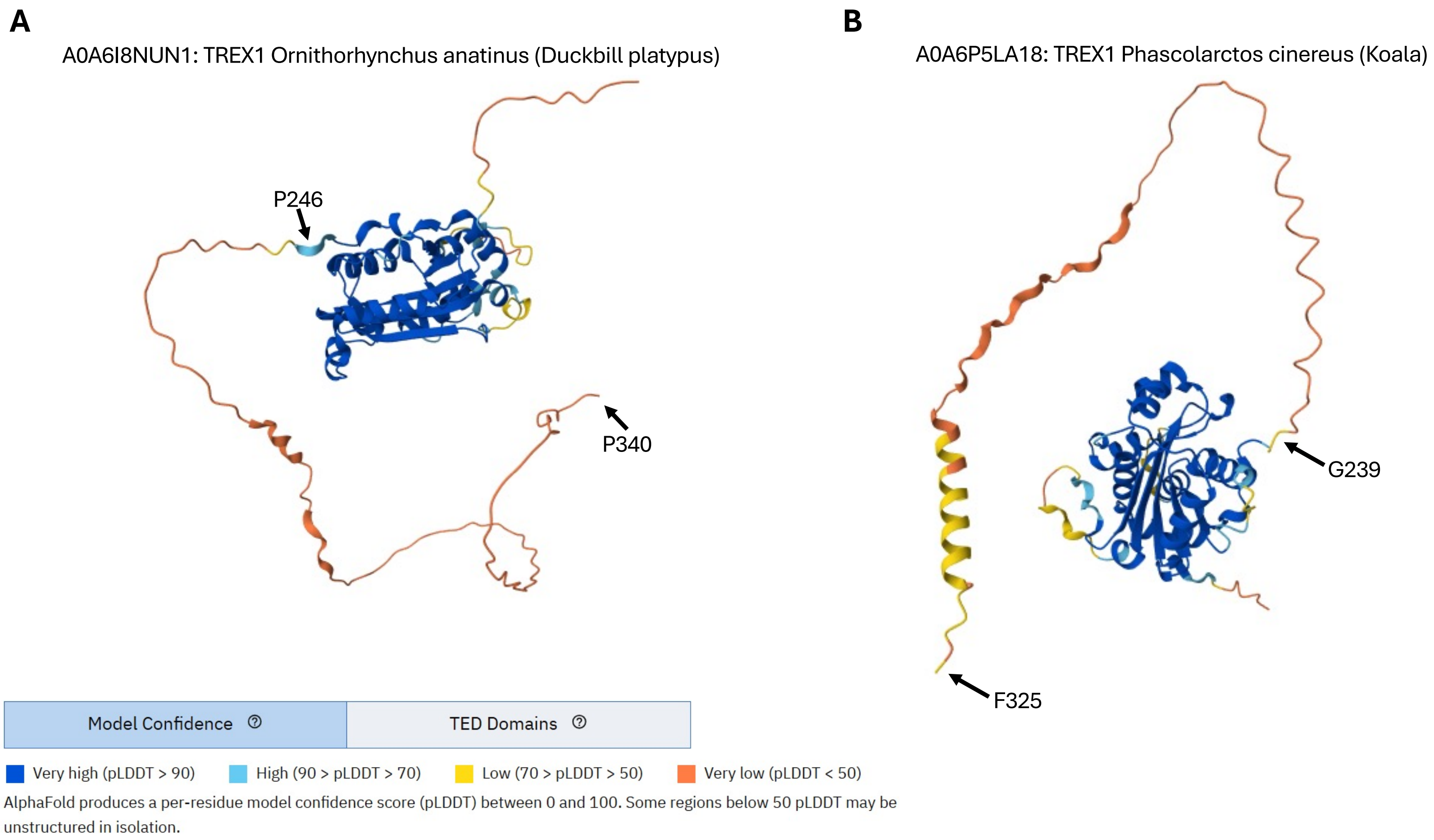

Fig. S6. Modeling of two TREX1 orthologs with distinct C-terminal sequences with AlphaFold. AlphaFold models of TREX1 from **(A)** duckbill platypus (UniProt ID: A0A6I8NUN1), and **(B)** koala (UniProt ID: A0A6P5LA18). Regions with low model confidence are displayed in yellow and orange, whereas regions with high model confidence appear in blue. The C-terminal domain of both TREX1 orthologs appear mostly disordered and these parts of the models have low confidence scores. Black arrows indicate start and end of C-terminal regions of interest (P246 to P340 in TREX1 from duckbill platypus, and G239 to F325 in TREX1 from koala).

A

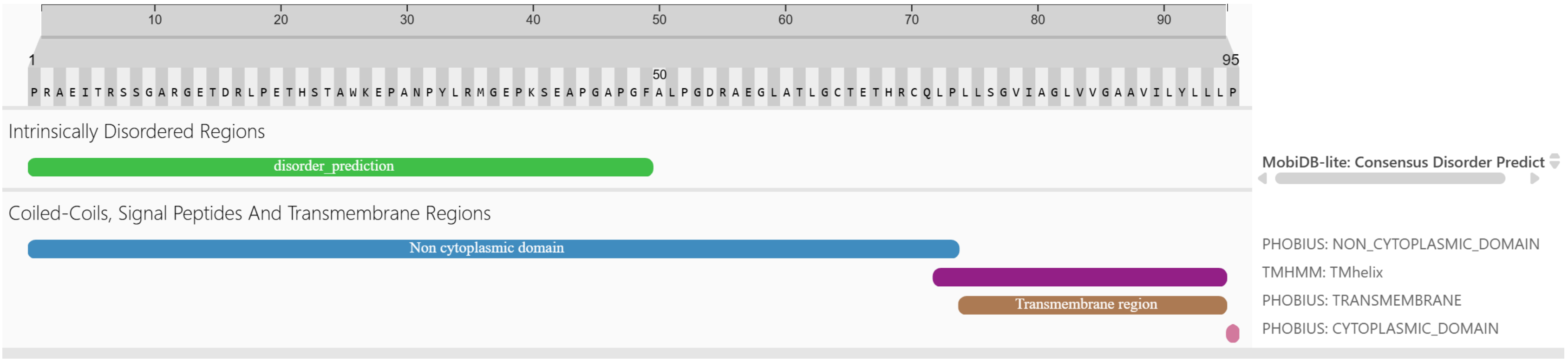

B

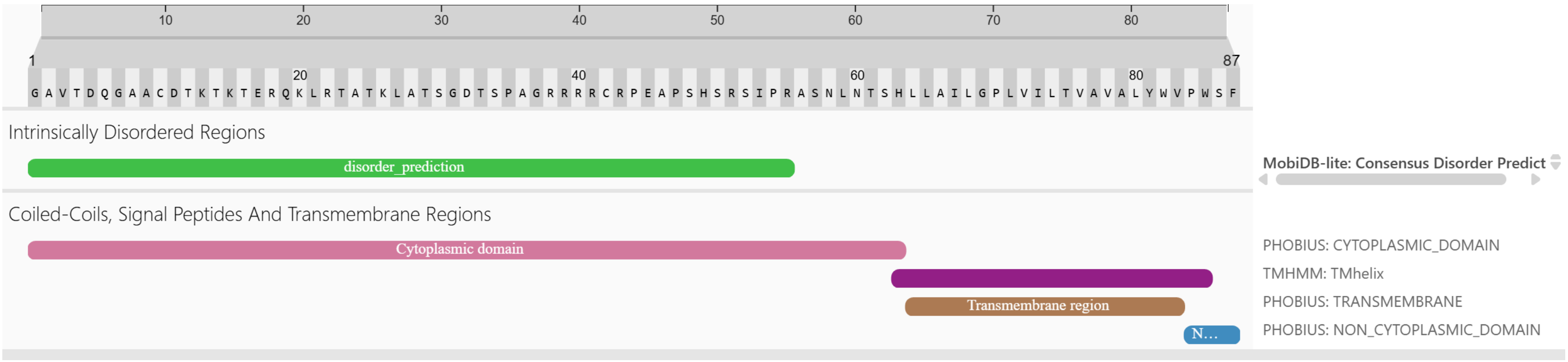

Fig. S7. Functional analysis of C-terminal sequences of platypus and koala TREX1 proteins with InterPro scan. The C-terminal sequences from **(A)** duckbill platypus (UniProt ID: A0A6I8NUN1, residues 246-340), and **(B)** koala (UniProt ID: A0A6P5LA18, residues 239-325) were submitted to the InterPro server to identify any functional domain. Both C-terminal regions contained a disordered region followed by a transmembrane helix, consistent with anchoring within a membrane.

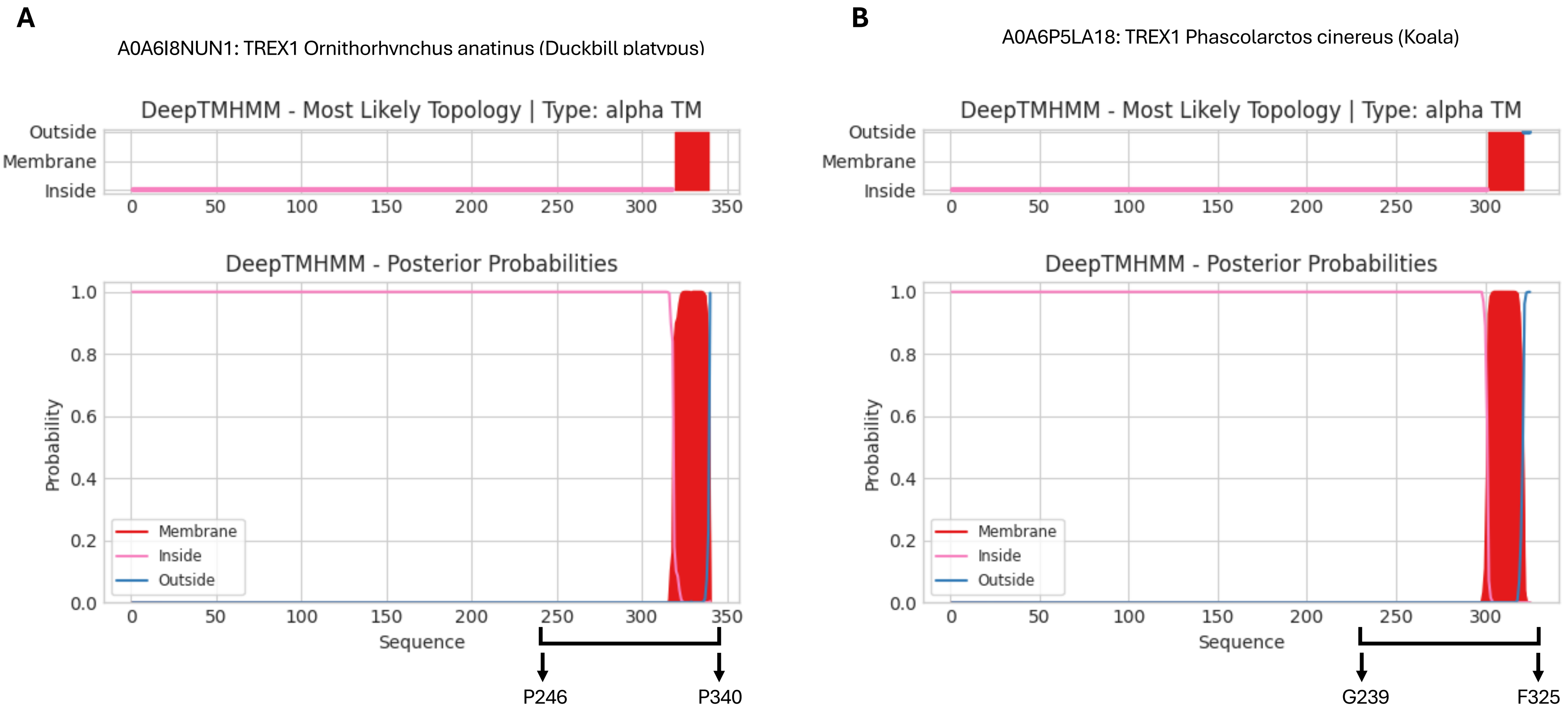

Fig. S8. Topology prediction of two TREX1 orthologs with distinct C-terminal sequences with DeepTMHMM. DeepTMHMM predicts a transmembrane section at the C-terminal of both **(A)** duckbill platypus (UniProt ID: A0A6I8NUN1) and **(B)** koala (UniProt ID: A0A6P5LA18). This prediction is consistent with the alpha-helix modeled at the C-terminal of the koala TREX1 protein by AlphaFold, and the presence of a transmembrane domain supports the anchoring of TREX1 within a membrane. Black arrows indicate start and end of C-terminal regions of interest (P246 to P340 in TREX1 from duckbill platypus, and G239 to F325 in TREX1 from koala).

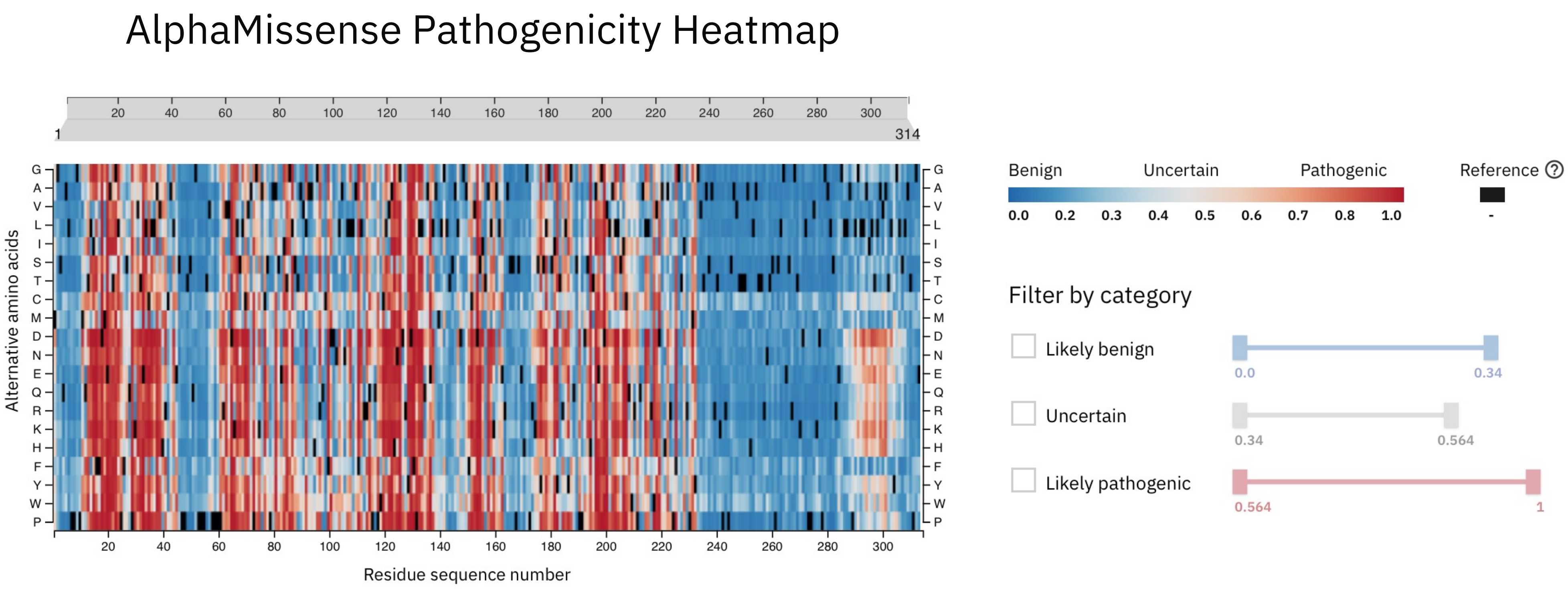

Fig. S9. Heatmap of predicted TREX1b AlphaMissense pathogenicity scores as provided by the AlphaFold server. The image shown corresponds to the entry AlphaFold entry AF-Q9NSU2-F1-v4 (https://alphafold.ebi.ac.uk/entry/Q9NSU2). **Reference** shows the amino acid residues in the UniProt reference protein sequence Q9NSU2, based on the hg38 version of human genome assembly.

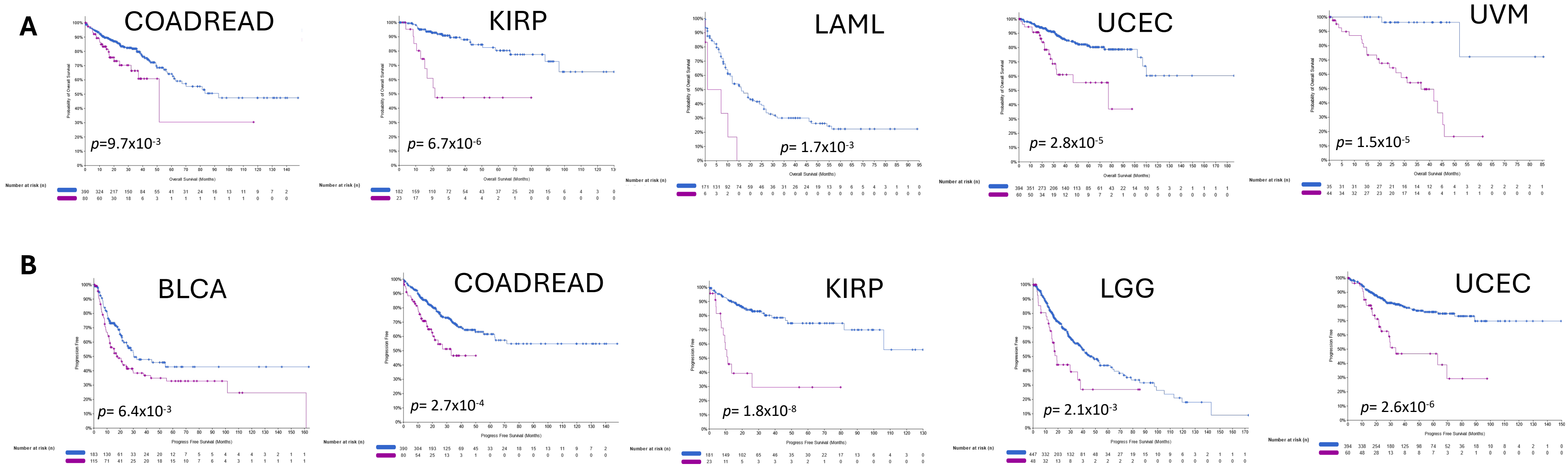

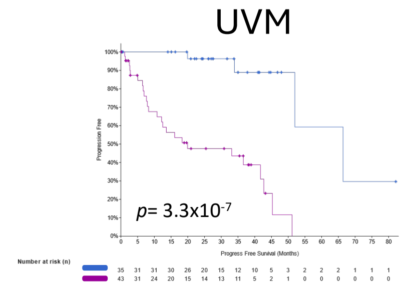

— TREX1 WT
— TREX1 loss

Fig. S10. *TREX1* survival impact in TCGA datasets. Kaplan-Meier survival curves of survival stratified by TREX1 status for **(A)** TCGA studies with significant association (p < 0.05) between *TREX1* loss and overall survival (data from cBioPortal), and **(B)** TCGA studies with significant association (p < 0.05) between *TREX1* loss and progression-free survival in TCGA datasets (data from cBioPortal). *TREX1* CNA status is stratified into two groups: unaltered (no mutation or CNA; blue) and *TREX1* deletion (single copy or deep deletion; purple). **BLCA:** bladder urothelial carcinoma, **COADREAD:** colorectal adenocarcinoma, **KIRP:** kidney renal papillary cell carcinoma, **LAML:** acute myeloid leukemia, **LGG:** brain lower grade glioma, **UCEC:** uterine corpus endometrial carcinoma, **UVM:** uveal melanoma.

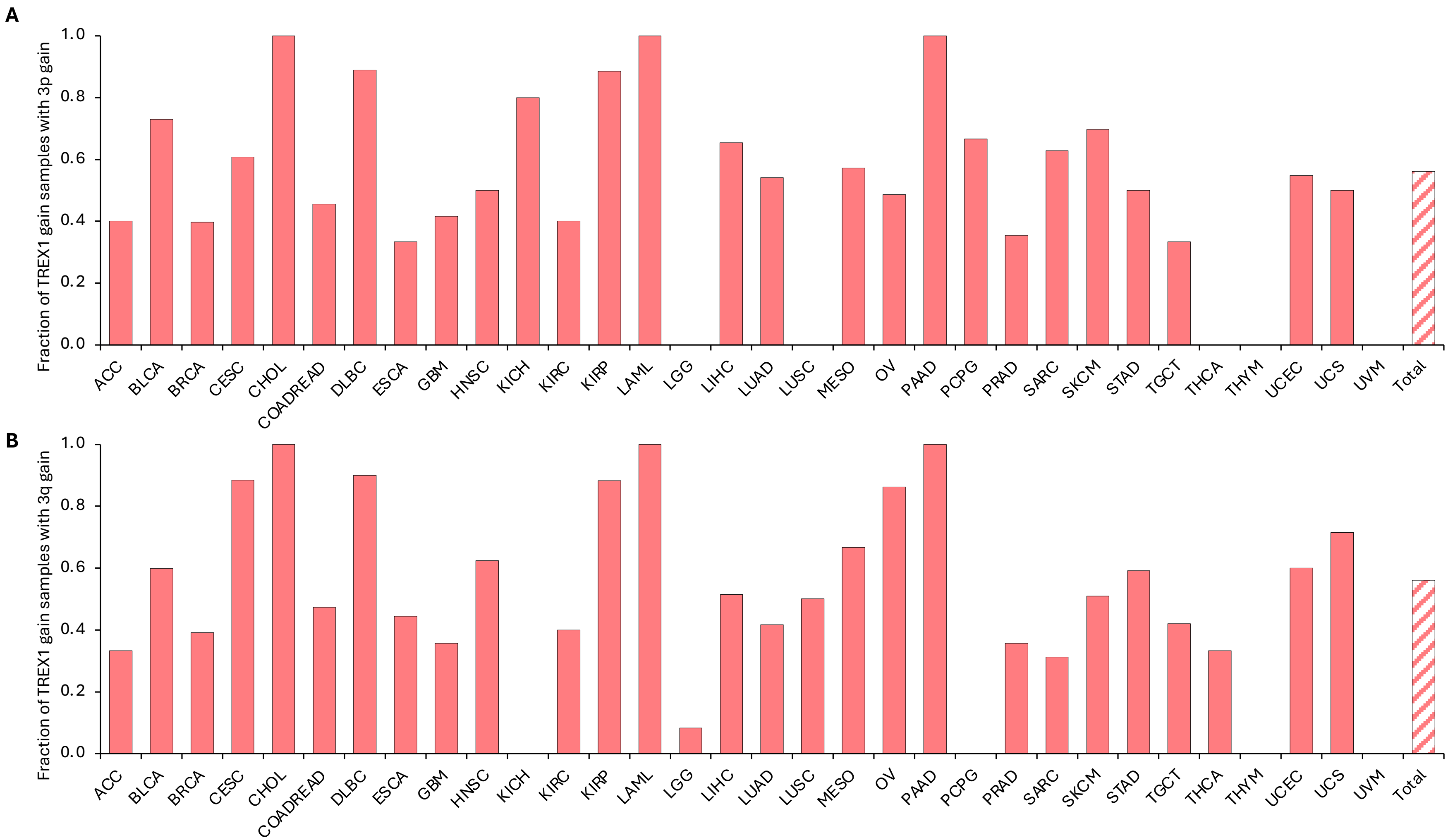

Fig. S11. Fraction of samples with co-incident copy number gain in *TREX1* and chromosome 3p or 3q. (**A**) Samples with TREX1 gain and co-incident 3p gain. (**B**) Samples with *TREX1* gain and co-incident 3q gain. Detailed number of samples per *TREX1* status and 3p or 3q status subgroups can be found in Tables S15 and S16.

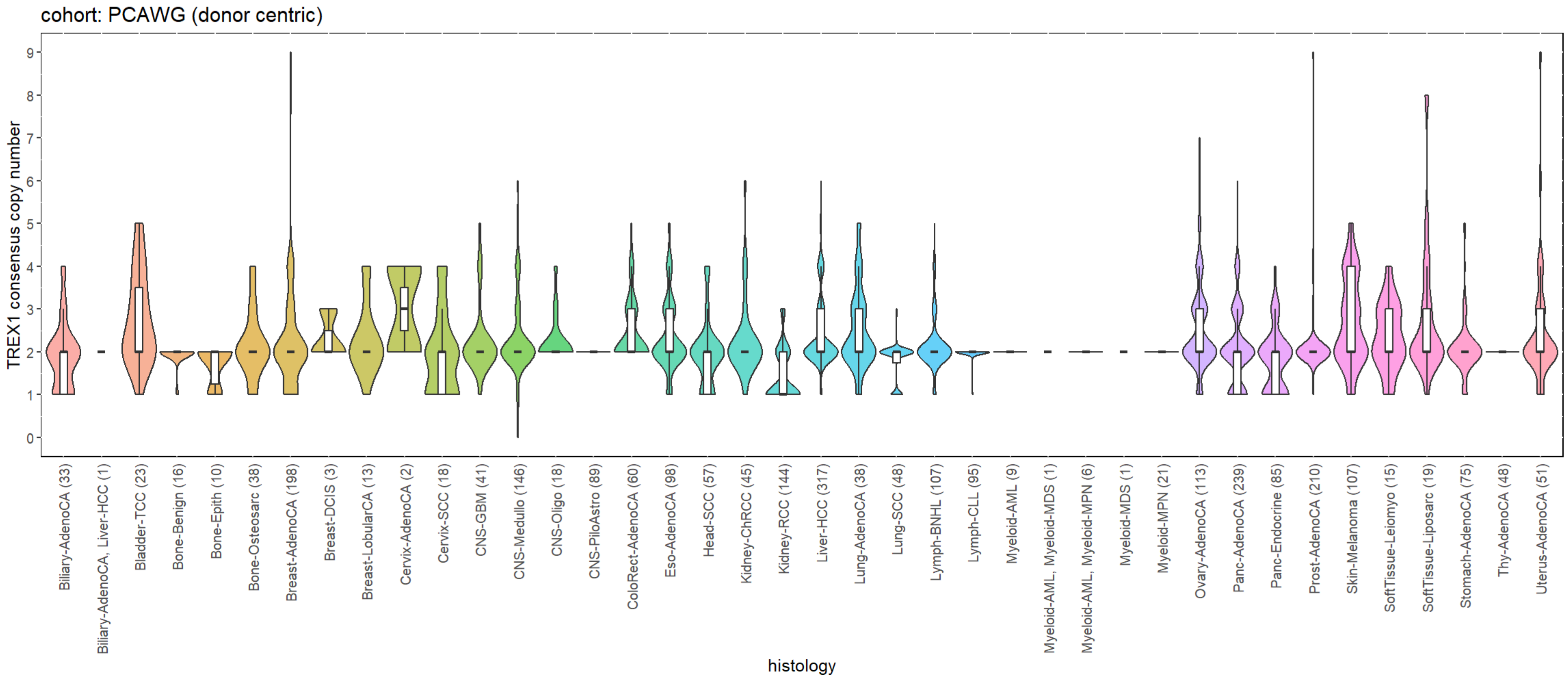

Fig. S12. Violin plots and boxplots showing *TREX1* copy number in the Pan-Cancer Analysis of Whole Genomes (PCAWG) dataset stratified by histology. The violins were scaled to have the same maximum width. Numbers in parenthesis indicate sample size for each tumor category. The CNA data were obtained from the PCAWG Xena hub [87,88] and exported using the UCSC Xena Browser [89]. Gene level *TREX1* copy number data were available for 2,658 samples in the PCAWG dataset.
